# Metabolomic and Lipidomic Profiling of Bone Marrow Plasma Differentiates Patients with Monoclonal Gammopathy of Undetermined Significance from Multiple Myeloma

**DOI:** 10.1038/s41598-020-67105-3

**Published:** 2020-06-24

**Authors:** Wilson I. Gonsalves, Katarzyna Broniowska, Erik Jessen, Xuan-Mai Petterson, Alexander Graham Bush, Jaimee Gransee, Martha Q. Lacy, Taro Hitosugi, Shaji K. Kumar

**Affiliations:** 1 The Division of Hematology, Mayo Clinic, Rochester, MN United States; 2 Biostatistics and Informatics, Mayo Clinic, Rochester, MN United States; 3 Endocrinology and the Department of Oncology, Mayo Clinic, Rochester, MN United States; 4 Molecular Therapeutics, Mayo Clinic, Rochester, MN United States; 5 Metabolon Inc, Morrisville, NC, Mayo Clinic, Rochester, MN United States

**Keywords:** Myeloma, Cancer metabolism

## Abstract

Oncogenic drivers of progression of monoclonal gammopathy of undetermined significance (MGUS) to multiple myeloma (MM) such as c-MYC have downstream effects on intracellular metabolic pathways of clonal plasma cells (PCs). Thus, extracellular environments such as the bone marrow (BM) plasma likely have unique metabolite profiles that differ from patients with MGUS compared to MM. This study utilized an untargeted metabolite and targeted complex lipid profiling of BM plasma to identify significant differences in the relative metabolite levels between patients with MGUS and MM from an exploratory cohort. This was followed by verification of some of the metabolite differences of interest by targeted quantification of the metabolites using isotopic internal standards in the exploratory cohort as well as an independent validation cohort. Significant differences were noted in the amino acid profiles such as decreased branch chain amino acids (BCAAs) and increased catabolism of tryptophan to the active kynurenine metabolite 3-hydroxy-kynurenine between patients with MGUS and MM. A decrease in the total levels of complex lipids such as phosphatidylethanolamines (PE), lactosylceramides (LCER) and phosphatidylinositols (PI) were also detected in the BM plasma samples from MM compared to MGUS patients. Thus, metabolite and complex lipid profiling of the BM plasma identifies differences in levels of metabolites and lipids between patients with MGUS and MM. This may provide insight into the possible differences of the intracellular metabolic pathways of their clonal PCs.

## Introduction

Multiple myeloma (MM) is a clonal plasma cell (PC) disorder characterized by the presence of end organ damage such as lytic bone disease, anemia, hypercalcemia or renal insufficiency^1^. It is always preceded by an asymptomatic, pre-cursor phase known as monoclonal gammopathy of undetermined significance (MGUS) that is typically monitored with yearly follow up only^2,3^. Several oncogenic drivers responsible for the progression of clonal PCs from an indolent MGUS phase to malignant MM have been identified such as MYC structural variants, activating mutations of RAS and NF-kB pathways, mutations of DIS3 or FAM46C etc^4^. However, most oncogenic drivers have downstream effects on various intracellular metabolic pathways^5^. As a result, dysregulated cellular metabolism is a hallmark of all malignancies since cancer cells adopt a distinct metabolic adaptation or phenotype to meet the augmented cellular demand for nucleotides, lipids, and amino acids created by increased rates of cellular proliferation^6^. Likewise, altered cellular metabolism also plays a role in the pathogenesis of MM^7^.

Since oncogenes like *MYC* play a major role in the pathogenesis of clonal PCs in MM^8^, there is likely a diverse amount of intracellular metabolic reprogramming that exists in clonal PCs upon their progression from MGUS to MM. As a result, we hypothesis that the corresponding extracellular metabolite profiles (in this case, the metabolite profile of the bone marrow (BM) plasma) should subsequently reflect the antecedent changes in the various intracellular metabolic pathways of the clonal PCs located within the BM. Metabolomics can play a key role in differentiating between different cellular metabolic phenotypes based on plasma and intracellular metabolite profiles^9^. Few prior studies have determined various relative differences in the peripheral blood serum or plasma and/or BM plasma metabolite profiles between MGUS and MM patients^10–14^. However, most studies suffer from their inability to utilize samples adequately collected and processed in a timely manner as required for metabolomics-based assessments^15^. Furthermore, none of the studies confirmed the actual concentration differences of metabolites of interest determined by the untargeted metabolomics assessment between the two groups. Thus, to overcome these aforementioned shortcomings, we analyzed the metabolite profile of BM plasma samples obtained prospectively in a manner suitable for metabolomics studies from patients with MGUS and MM undergoing clinical BM aspirations. We utilized untargeted metabolite profiling to identify significant relative differences in metabolite levels between the two-groups followed by verification of these differences by targeted quantification of the metabolites of interest. We then validated the presence of these quantitative metabolite differences in a set of 25 prospective patients with either MGUS, active MM or MM with no evidence of disease or in a complete remission after therapy.

## Results

### Clinical and laboratory characteristics of patients with MM and MGUS

Prospective BM plasma samples were obtained from 25 consecutive MGUS patients and 25 consecutive MM patients (16 with newly diagnosed MM (NDMM) and 9 with relapsed and/or refractory MM (RRMM)) who were undergoing BM aspirations as part of their routine clinical care. These two groups of patients were categorized as the exploratory set. The clinical and pathological characteristics of these patients are summarized in Supplementary Table 1. As expected, the MGUS patients had a higher median hemoglobin level, lower median BMPC percentage and lower median serum M-spike compared to patients with MM. Among patients with MM, those with RRMM had a higher proliferation rate as measured by the S-phase percentage than those with NDMM.

### Global metabolite profiling of marrow plasma from exploratory set of MGUS and MM patients

**Figure 1:**
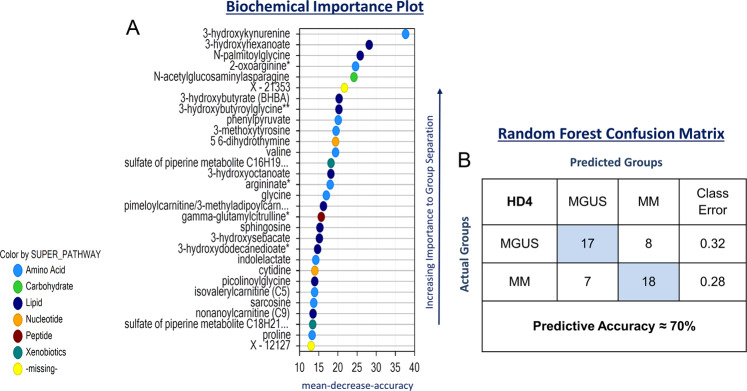
(**A**) Random forest analysis of bone marrow plasma samples from patients with MGUS (N = 25) and MM (N = 25) based on relative levels of metabolites depicting top 30 metabolites responsible for group separation between MGUS and MM. (**B**) Random forest confusion matrix demonstrating the performance of the model generated by the random forest analysis of metabolites in accurately predicting MM and MGUS.

A total of 1,088 biochemicals were detected by an untargeted ultra-performance liquid chromatography tandem mass spectrometer (UPLC-MS/MS) in both groups of samples of which 823 were compounds of known structural identity and the remaining 265 compounds were unnamed biochemicals (compounds without a library entry, designated with X-). The relative concentration of a 198 of these aforementioned biochemical were significantly different between the two groups with 110 increased and 88 decreased in the MM group compared to the MGUS group. Supplementary Fig. 1 depicts the hierarchical clustering analysis of this cohort which shows sub-clustering based on MGUS vs. MM disease status. Random forest analysis on the differences in the metabolite profiles between MGUS and MM BM plasma samples was conducted and Fig. 1A demonstrated the top 30 metabolite species contributing to the group separation which was composed of several amino acid and fatty acid metabolites. However, this resulted in a model with a moderate predictive accuracy of 70% (random chance for two groups would be expected to yield 50%) (Fig. 1B). Furthermore, this model had a sensitivity of 72% and a specificity of 68% in detecting the presence of MM. Receiver operating characteristic (ROC) curves were generated on the top four metabolites contributing to the group separation (3-hydroxykynurenine, 3-hydroxyhexanoate, N-palmitoylglycine and 2-oxoarginine) to assess their potential as individual biomarkers for MM (Supplementary Fig. 2).

Among amino acids, the levels of BCAAs such as valine, isoleucine, and leucine, together with their respective keto-acids, 3-methyl-2-oxobutyrate, 3-methyl-2-osovalerate, and 4-methyl-2-oxopentanoate, were lower in the MM group relative to MGUS. There were also diminished levels of tyrosine and higher levels of several tyrosine-derived metabolites such as 3-methoxytyrosine, homovanillate (HVA), and vanillic alcohol sulfate in the MM group relative to MGUS. Similar differences were also noted in tryptophan metabolism where tryptophan and serotonin declined, while 3-hydroxykynurenine and 8-methoxykynurenate accumulated in the MM group compared to MGUS. Among nucleotides, there were marked differences noted such as increased levels of several purine (e.g. N1-methyladensoine) and pyrimidine (e.g. pseudouridine 5,6-dihydrouridine) intermediates whereas some nucleotide metabolites were also depleted (e.g. 2-deoxyuridine, 5,6-dihydrothymine) in comparing the MM group vs MGUS. Changes were also noted in several amino sugar metabolites, including N-acetylglucosaminylasparagine, glucuronate, erythronate and N-acetylglucosamine/N-acetylgalactosamine between the MM and MGUS groups. Among fatty acid oxidation metabolites, there were lower levels of beta-oxidation intermediates, including medium and very long-chain acylcarnitines in the MM group relative to MGUS. These aforementioned differences were accompanied by declines in free carnitine and accumulation of ketone bodies, acetoacetate and 3-hydroxybutyrate (BHBA) in the MM group when compared to MGUS. Supplementary Table 2 provides information on the fold change differences of the 1,088 biochemicals identified between the two groups. A metabolite set enrichment analysis (MSEA) assessment as shown in Supplementary Fig. 3 also supported the presence of alterations in various amino acid metabolic pathways and the pyrimidine metabolic pathway in MM compared to MGUS.

### Quantitative lipid profiling comparing marrow plasma from exploratory set of MGUS vs. MM patients

**Figure 2:**
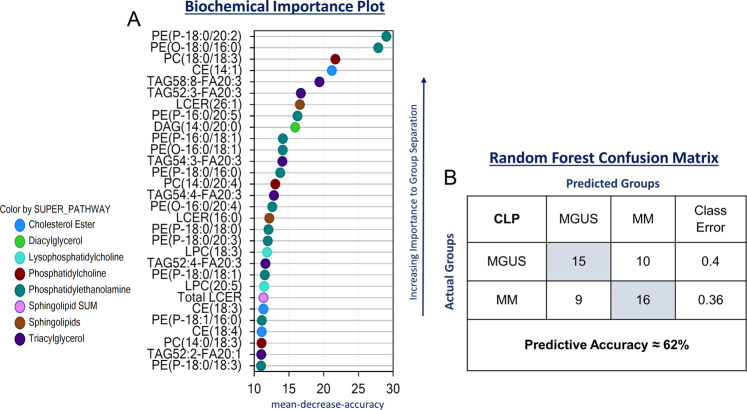
(**A**) Random forest analysis of bone marrow plasma samples from patients with MGUS (N = 25) and MM (N = 25) based on concentrations of complex lipids depicting top 30 complex lipids responsible for group separation between MGUS and MM. (**B**) Random forest confusion matrix demonstrating the performance of the model generated by the random forest analysis of complex lipids in accurately predicting MM and MGUS.

**Figure 3:**
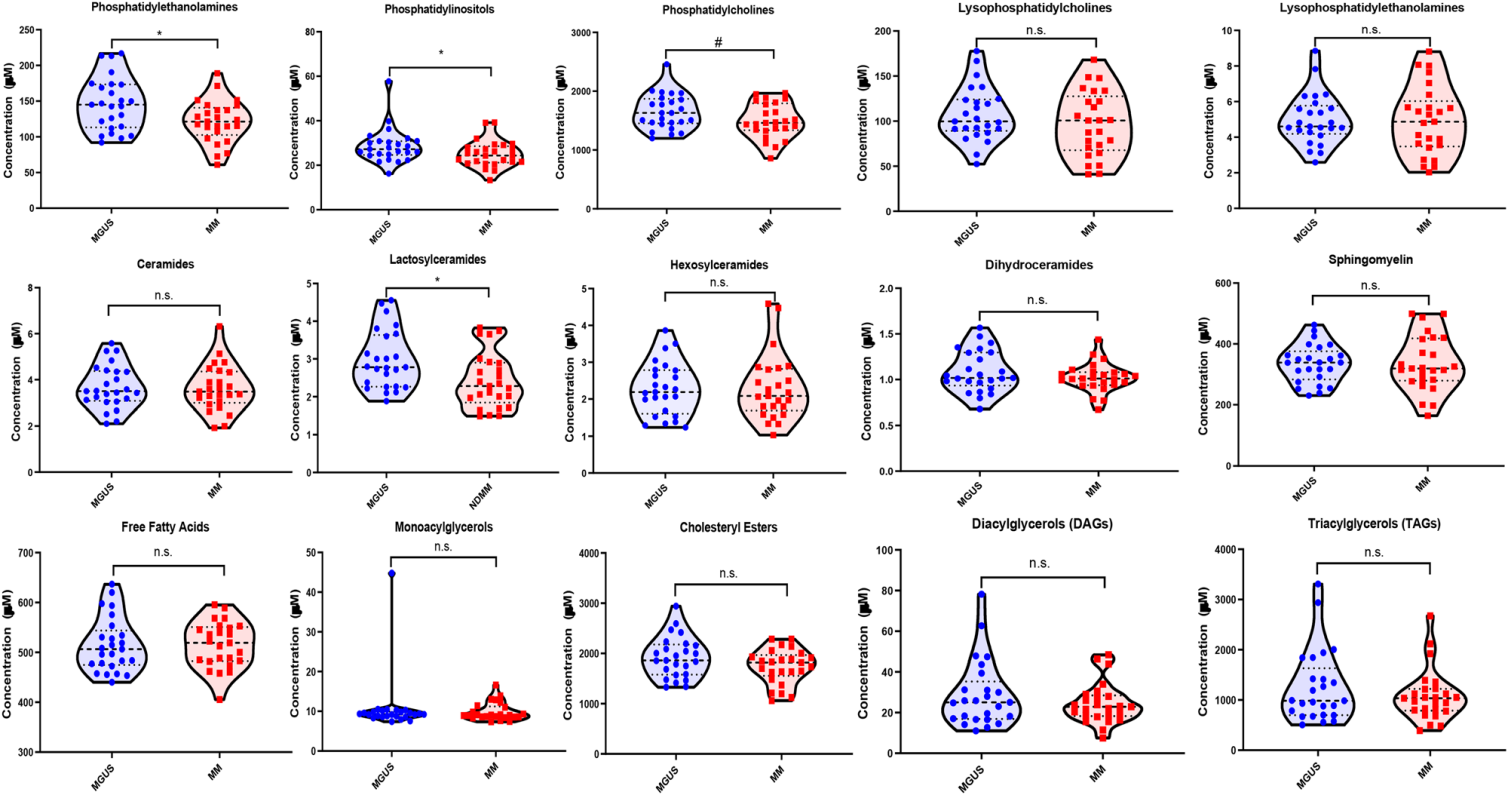
Violin plots comparing the median concentrations of different total species of complex lipids in the bone marrow plasma between MGUS (N = 25) and MM (N = 25) groups. Data was analyzed by Mann-Whitney U test where ***p < 0.001, **p < 0.01, *p < 0.05, ^#^p < 0.1 but > 0.05, n.s. non-significant.

Comprehensive complex lipid panel analysis in the BM plasma of the exploratory MGUS and MM groups was performed to determine if there were differences. There was a total of 189 biochemicals that were significantly different between the two groups with 4 increased and 185 decreased in the MM group compared to the MGUS group. Supplementary Fig. 4 depicts the hierarchical clustering analysis of this cohort which shows sub-clustering based on MGUS vs. MM disease status. Random forest analysis on the differences in the lipid profiles between MGUS and MM BM plasma samples was conducted and the biochemical importance plot depicting the top 30 complex lipid species contributing to the group separation in Fig. 2A demonstrated that the top four major discriminatory lipids were phosphatidylethanolamines (n = 2), phosphatidylcholines (n = 1) and a cholesterol ester. This resulted in a model with a moderate predictive accuracy of 62% (random chance for two groups would be expected to yield 50%) (Fig. 2B). Furthermore, this model had a sensitivity of 64% and a specificity of 60% in detecting the presence of MM. Receiver operating characteristic (ROC) curves were generated on the top four lipids contributing to the group separation (PE(P-18:0/20:2), PE(O-18:0/16:0), PC(18:0/18:3) and CE(14:1)) to assess their potential as individual biomarkers for MM (Supplementary Fig. 5). In terms of lipid classes, lower levels of total PEs (p = 0.027), lactosylceramides (LCER) (p = 0.011) and phosphoinositols (PI) (p = 0.047) were detected in samples collected from MM as compared to MGUS with a similar trend in total phosphorylcholines (PC) (p = 0.09) **(**Fig. 3**)**. Supplementary Table 3 provides information on the fold change differences of all the complex lipid species identified between the two groups.

### Quantitative verification of differences in the amino acids and their metabolites between the marrow plasma from the exploratory set of MGUS vs. MM patients

**Figure 4:**
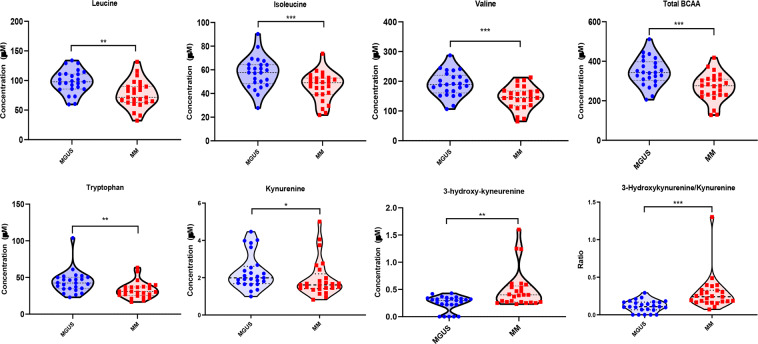
Violin plots comparing the median concentrations of different branched chain amino acid (BCAAs) as well as tryptophan and its catabolites in the bone marrow plasma between MGUS (N = 25) and MM (N = 25) groups. Data was analyzed by Mann-Whitney U test where ***p < 0.001, **p < 0.01, *p < 0.05, ^#^p < 0.1 but >0.05, n.s. non-significant.

**Figure 5:**
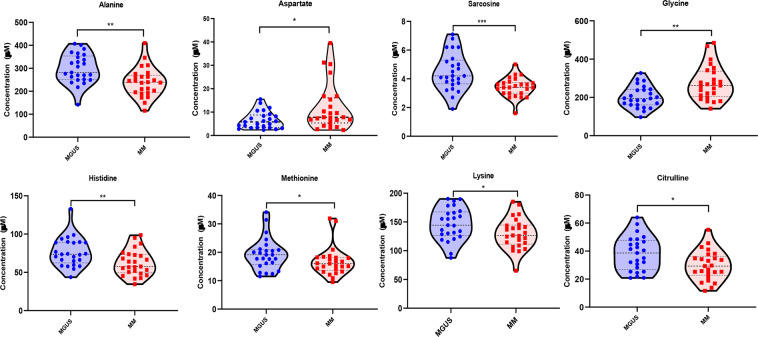
Violin plots comparing the median concentrations of various amino acids and metabolites in the bone marrow plasma between MGUS (N = 25) and MM (N = 25) groups. Data was analyzed by Mann-Whitney U test where ***p < 0.001, **p < 0.01, *p < 0.05, ^#^p < 0.1 but >0.05, n.s. non-significant.

Given several relative differences observed in amino acid profiles between the exploratory MGUS and MM groups, we then performed absolute quantification of the concentrations of amino acids and their metabolites in the BM plasma of the same exploratory MGUS and MM groups to verify the relative differences observed in the untargeted analyses. The concentrations of the total BCAA (p = 0.0003) and their individual components such as valine (p = 0.0003), leucine (p = 0.001) and isoleucine (p = 0.0002) were significantly lower in the MM group compared to the MGUS group as indicated by the untargeted analysis **(**Fig. 4**)**. Similarly, we detected lower concentration levels of tryptophan (p = 0.002) and its downstream metabolite, kynurenine (p = 0.029), in the BM plasma of the MM group compared to MGUS with a resultant higher 3-hydroxy-kynurenine concentration level (p = 0.003) in the MM group compared to MGUS **(**Fig. 4**)**. As a result, the ratio of 3-hydroxy-kynurenine to kynurenine was also significantly higher in the MM group compared to the MGUS group (p < 0.0001). The average concentrations of various other amino acids and their metabolites were also lower in the MM group such as alanine (p = 0.001), sarcosine (p = 0.008), histidine (p = 0.005), methionine (p = 0.037), lysine (p = 0.041) and citrulline (p = 0.019) compared to the MGUS group **(**Fig. 5**)**. In contrast, the average concentrations of glycine (p = 0.002) and aspartate (p = 0.049) was higher in the MM group compared to that of the MGUS group.

### Validation of quantitative differences among amino acids and their metabolites in the marrow plasma from MGUS vs. MM patients

**Figure 6:**
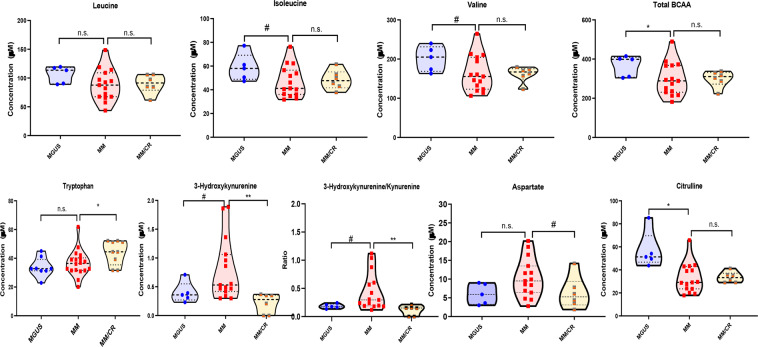
Violin plots comparing the median concentrations of various amino acids and metabolites in the bone marrow plasma between MGUS (N = 5), MM (N = 15) and MM in CR (N = 6) groups. Data was analyzed by Mann-Whitney U test where ***p < 0.001, **p < 0.01, *p < 0.05, ^#^p < 0.1 but >0.05, n.s. non-significant.

The quantification was also performed in a different validation set of 25 prospective patients with plasma cell disorders ranging from MGUS (N = 5) and MM (N = 20 of which 6 were in a complete remission and 14 were patients with either newly diagnosed or relapsed MM). Again, it was demonstrated that the median concentration of the total BCAA was significantly lower in the MM group compared to the MGUS group (p = 0.033) **(**Fig. 6**)**. In contrast, among the individual components of BCAAs such as valine, leucine and isoleucine, there was no observed difference in the concentration between the MM and MGUS group. Surprisingly, even though the median concentration of 3-hydroxy-kynurenine (p = 0.077) and the ratio of 3-hydroxy-kynurenine to kynurenine (p = 0.05) appeared numerically higher in the BM plasma of MM compared to MGUS, this was not statistically significant. Interestingly, those MM patients who were in a complete remission (CR) after therapy had lower median 3-hydroxy-kynurenine (p = 0.003) and ratio of 3-hydroxy-kynurenine to kynurenine (p = 0.002) levels compared to those with active MM but similar to the median levels of the MGUS. Finally, in this validation set, it was noted that the median concentration of aspartate was higher in the MM group compared to the group of MM patients in a CR (p = 0.079) but this was not statistically significant **(**Fig. 6**)**.

### Correlation of metabolite quantities in the marrow plasma to the percentage of bone marrow plasma cells and proliferation index and cytogenetics

**Figure 7:**
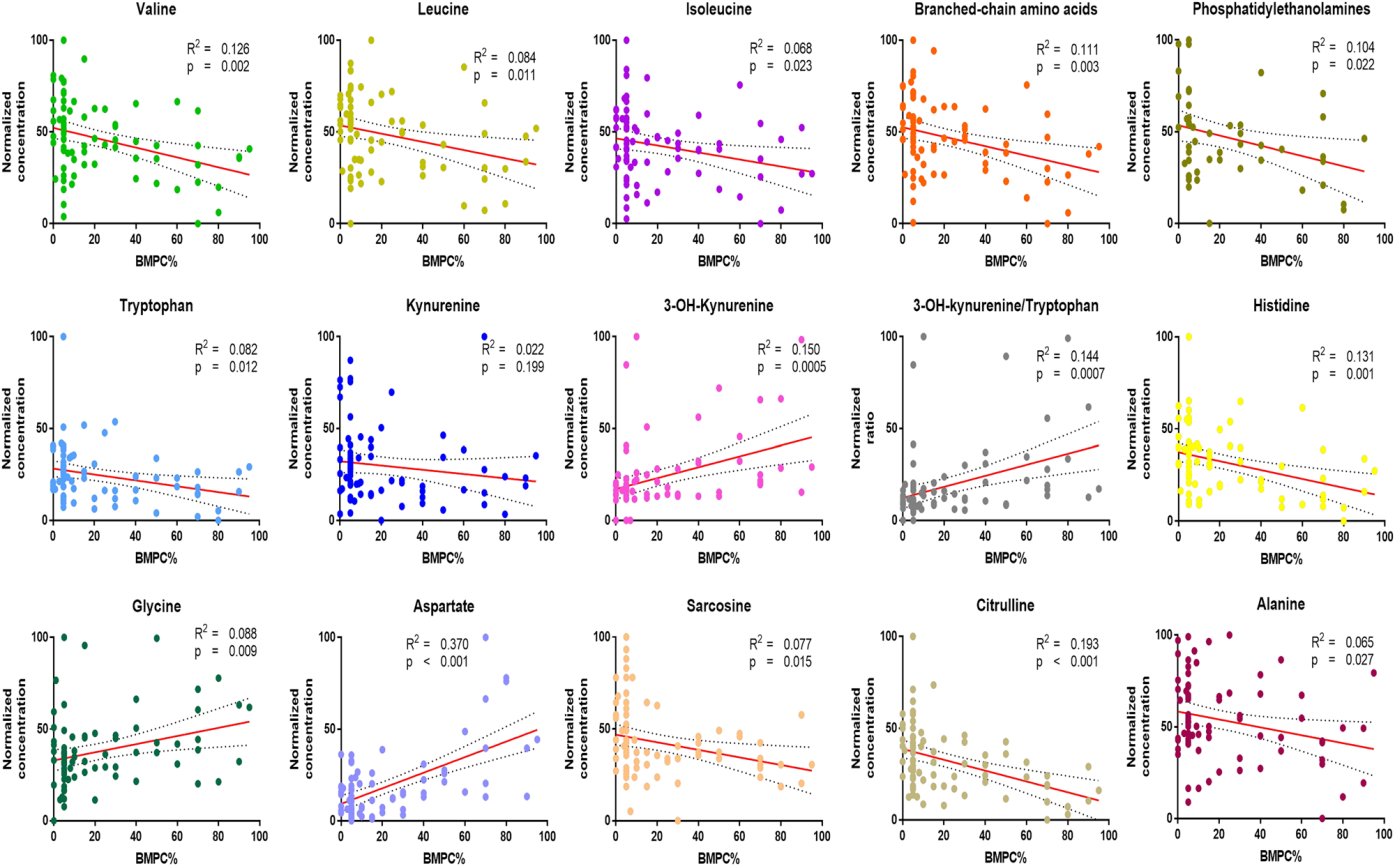
XY-correlation plots comparing the normalized concentrations of various amino acids and metabolites in the bone marrow plasma of patients with MGUS and MM and the percentage of clonal PCs in their bone marrow.

**Figure 8:**
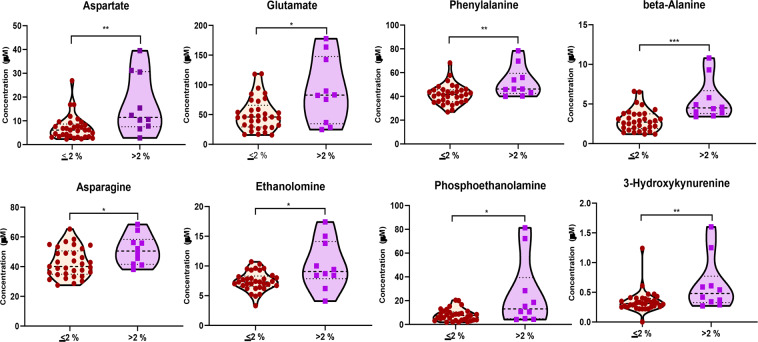
Violin plots comparing the median concentrations of various amino acids and metabolites in the bone marrow plasma from MGUS and MM patients based on whether the S-phase of their cPCs were low (<2%) (N = 33) or high (>2%) (N = 10). Data was analyzed by Mann-Whitney U test where ***p < 0.001, **p < 0.01, *p < 0.05, ^#^p < 0.1 but >0.05, n.s. non-significant.

To determine if the changes in the concentrations of the various amino acid and lipid metabolites are directly related the clonal plasma cells in the BM, we sought to determine if there was a direct positive or negative correlation between the absolute concentration of these metabolites with the percentage of clonal PCs present at the time of the BM aspiration. As shown in Fig. 7, we observed a positive correlation between the concentrations of glycine, aspartate and 3-hydroxy-kynurenine and the number of clonal PCs present in the marrow. In contrast, there was a negative correlation between the concentrations of the various BCAAs, tryptophan, kynurenine, histidine, sarcosine, citrulline, alanine and phosphoethanolamine and the number of clonal PCs in the marrow. We did not observe a difference between the concentrations of the various amino acid and complex lipids with respect to their primary cytogenetic abnormalities (hyperdiploid vs. non-hyperdiploid). Finally, there appeared to be a direct correlation with the level of several metabolites such as aspartate (p = 0.005), asparagine (p = 0.027), ethanolamine (p = 0.027), phosphoethanolamine (p = 0.027), glutamate (p = 0.041), beta-Alanine (p = 0.0002), phenylalanine (p = 0.008) and 3-hydroxy-kynurenine (p = 0.008) in BM plasma of MGUS and MM patients and the proliferation rate (S-phase %) of their clonal PCs in the BM **(**Fig. 8**)**.

## Discussion

The current study demonstrates that the BM plasma concentration levels of select complex lipids, amino acids and their corresponding metabolites are significantly different between patients diagnosed with MGUS compared to MM. We specifically noted significant differences in the amino acid levels such as decreased branch chain amino acids (BCAAs) and the increased catabolism of tryptophan to the active kynurenine metabolite 3-hydroxy-kynurenine between patients with MGUS and MM. There was also a decrease in the total levels of phosphatidylethanolamines (PE), lactosylceramides (LCER) and phosphoinositols (PI) in the BM plasma samples from MM compared to MGUS patients. These differences reflect several of the expected intracellular metabolism changes that take place within cPCs upon progression from a pre-malignant MGUS phase to a malignant MM phase. Furthermore, our study demonstrates that some of these aforementioned metabolite levels in the BM plasma are dependent on the degree of cPCs present in the BM and their proliferation rate. Whereas the levels of some of these metabolites in patients with MM can revert to that of MGUS based upon effective response to therapies (achieving CR).

The BCAAs such as valine, isoleucine, and leucine are significant components of all human proteins. However, BCAAs can also be degraded to supply carbon substrate for various anabolic cellular (i.e. gluconeogenesis or fatty acid synthesis) or energy (i.e. the TCA cycle) pathways. The branched-chain aminotransferase (BCAT) is responsible for converting BCAAs to their respective alpha-keto acid derivatives which is then further metabolized in the mitochondria via the branched-chain alpha-keto acid dehydrogenase (BCKD) complex. In our study, the global metabolite profiling determined that the levels of valine, isoleucine, and leucine, together with their respective keto-acids, 3-methyl-2-oxobutyrate, 3-methyl-2-osovalerate, and 4-methyl-2-oxopentanoate, were lower in the BM plasma of MM subjects relative to the MGUS group (Supplementary Table 2). Furthermore, these differences in the total concentrations of BCAAs were verified by targeted quantification in both discovery **(**Fig. 4**)** and validation **(**Fig. 6**)** sets of MM and MGUS samples evaluated. The declines in BCAAs could point towards increased utilization of BCAAs by the clonal PCs in the BM as energetic and/or biosynthetic substrates. This has been noted in a prior untargeted metabolomics study where lower levels of BCAAs in peripheral blood plasma were reported in NDMM patients as compared to healthy control individuals^11^.

Difference were also noted in tryptophan metabolism in both discovery and validation sets of MM and MGUS patients where the concentration levels of tryptophan metabolites such as 3-hydroxykynurenine was higher for the MM compared to the MGUS group. These higher levels of 3-hydroxy-kynurenine could reflect a higher activation of indoleamine 2,3-dioxygenase 1 (IDO1) in the kynurenine pathway within the clonal PCs of MM compared to MGUS. This increased activation of IDO1 has been reported in many other human tumors in order to evade immune response^16^ and increased IDO expression is a known independent prognostic variable for reduced overall survival in cancer patients^17^. Furthermore, the lower levels of serotonin in MM cohort could be secondary to the redirection of tryptophan towards kynurenine pathway. Kynurenine and its metabolites can exert immunosuppressive effects through the aryl hydrocarbon receptor (AHR), leading to suppressed effector T cell proliferation and enhanced regulatory T cell generation. Thus, tryptophan appears to be shunted to the kynurenine pathway and one possible explanation could be to support the immune evasion of the clonal PCs in MM. Recently, it has been demonstrated that the oncogene MYC is capable of increasing the expression of the tryptophan transporters SLC7A5 and SLC1A5 as well as the enzyme arylformamidase (AFMID) which is involved in the conversion of tryptophan into kynurenine^18^. Since c-Myc is a known driver of progression of clonal PCs in MGUS to MM, it is possible that the differences in the tryptophan-kynurenine metabolic pathway are a manifestation of one of many consequences of c-Myc activation. In a large-scale analysis of the intra and extracellular metabolite profiles of various cancer cell lines from the Cancer Cell Line Encyclopedia (CCLE), a wide range of intracellular kynurenine concentrations were noted across multiple cancer types with higher intracellular kynurenine levels being strongly associated with greater secretion into the spent media^19^. In addition, it was observed that the abundant intracellular kynurenine in cell lines were driven by either IDO1 or TDO or both being simultaneously expressed suggesting that cancer cells can probably use either enzyme or potentially both to produce kynurenine and its downstream metabolites^19^.

This untargeted metabolomics assessment in the exploratory set also identified increased levels of several purines and pyrimidine intermediates with concurrent reduction in the levels of some nucleotide metabolites within the BM plasma of MM in comparison to MGUS. These changes possibly indicate higher rates of DNA and RNA synthesis in the MM clonal PCs compared to MGUS needed to support the higher proliferation of clonal PCs in the BM of MM patients. These findings are further supported by declines in the concentration levels of sarcosine and augmentations in glycine levels detected in BM plasma of MM patients compared to that of MGUS as these biochemicals are involved in one-carbon group metabolism, which through tetrahydrofolate support purine biosynthesis and dTMP formation. These findings have been described by prior studies where the pyrimidine, pseudouridine, has been known to be in circulation in the blood at a higher level in MM patients and is an indirect measure of tRNA turnover, since it is generated from degradation of tRNA splicing intermediates^20^. Other levels of amino acids and metabolites in the BM plasma were also found to be associated with increased proliferation (S-phase >2%) of their clonal PCs in the BM **(**Fig. 7**)** such as aspartate, asparagine, ethanolamine, phosphoethanolamine, glutamate, beta-Alanine, phenylalanine and 3-hydroxy-kynurenine.

There were also significant differences noted in several aminosugar metabolites, including N-acetylglucosaminylasparagine, glucuronate, erythronate and N-acetylglucosamine/N-acetylgalactosamine between MM and MGUS patients in the exploratory set via untargeted metabolomics assessments. These compounds serve as substrates for glycosylation reactions, which are critical for folding and secretion of proteins, including cell surface receptors and ultrastructural components (e.g. extracellular matrix, ECM). This could imply the presence of significant remodeling of the bone marrow ECM of patients with MM which forms a permissive tumor microenvironment capable of progression of precursor PC disorders to MM^21^. There were also increases in dimethylarginine, derived from the catabolism of proteins containing methylated arginine residues, and in N-formylmethionine, a marker of mitochondrial protein turnover in the BM plasma of the MM group compared to that of MGUS. Collectively, these changes suggest alterations in protein turnover in bone marrow plasma of MM patients, which may reflect higher rates of cell proliferation and ECM remodeling in MM compared to MGUS patients.

Our results also indicated the fatty acid utilization pattern was different between MM and MGUS. For one, there were significant differences noted in several acylcarnitines between MM and MGUS patients in the exploratory set via untargeted metabolomics assessments. These declines in acylcarnitines, which are in equilibrium with their CoA derivatives, could be indicative of diminished beta-oxidative capacity which would be consistent with redirection of carbons towards lipid biosynthesis in rapidly proliferating cells. Secondly, there was accumulation of ketone bodies at a higher level in the BM plasma of MM compared to MGUS patients which could be caused by either increased fatty acid oxidation by MM clonal PCs or failure of these clonal PCs to effectively incorporate these ketones into the TCA cycle to support mitochondrial energetics. Finally, from a complex lipid standpoint, the PCs, PEs and PIs are important structural components of cell membranes and lower levels of these biochemicals in the BM plasma of the MM group compared to the MGUS group may suggest an increased utilization of these compounds towards membrane biosynthesis in samples collected from MM patients as those clonal PCs are more rapidly proliferating. Overall, this study demonstrates that metabolomic and lipidomic profiling can be exploited in the future to identify and validate biomarkers of clinical value in select patients with plasma cell disorders. For example, the entity known as smoldering multiple myeloma (SMM), which is a more advanced pre-cursor disorder to MM than MGUS, is a disease state wherein patients do not have any clinical symptoms of MM but have a higher amount of clonal PCs in their bone marrow (>10% but less than 60%) compared MGUS (defined by less than 10%)^22^. As a result, SMM patients have a variable risk of progression to MM where some SMM patients develop MM within the first 2 years of diagnosis as they likely had already started to biologically progress to MM whereas other SMM patients never develop MM even after 10 years of close follow-up similar to that observed in MGUS patients^23^. In the future, metabolite and lipid biomarkers in the BM plasma may be able to prognosticate which SMM patients have more of a MM phenotype rather than a MGUS phenotype since the former type of SMM patients may benefit in undergoing early systemic therapy to prevent the development of symptoms of MM.

Although further validation of the results using additional patient samples is necessary to increase the robustness of this analysis, this study demonstrates that the BM plasma samples obtained from patients diagnosed with MM and MGUS differed in metabolites associated with phospholipid metabolism, branched-chain and aromatic amino acids catabolism and nucleotide turnover. Nevertheless, there are several limitations to this study. First, we were unable to validate the concentration differences for several of the metabolites determined to be different between the MGUS and MM group of the exploratory set by the untargeted metabolomics assay. This is because we did not have authentic standards for each metabolite of interest. However, we were able to confidently quantify the concentrations of various amino acids and their metabolites in the BM plasma of patients in both the exploratory and validation sets. Second, the differences in the extracellular metabolite profile determined in the BM plasma between the MM and MGUS groups may not always represent the true differences in the intracellular metabolic pathways of the clonal PCs between the two groups. However, it provides a starting point for additional studies to assess potential targeting of intracellular metabolic pathways of clonal PCs for therapeutic purposes. Most importantly, our study lacks the presence of a control group, either healthy age-matched participants or age-matched patients with no evidence of a plasma cell dyscrasia due to our inability to have them undergo voluntary bone marrow aspirations to obtain marrow plasma samples. As a result, the metabolite differences observed between MGUS and MM are not proven to be specific for these disease entities and should not be considered as such. In the future, performing prospective studies that follow the peripheral blood and BM plasma metabolite profiles of MGUS and NDMM patients longitudinally in order to determine the dynamic metabolic changes associated with the progression from MGUS to MM will be important. This could provide a mechanistic viewpoint on the changes in the intracellular metabolism of clonal PCs associated in the context of the pathogenesis of MM.

## Methods

All methods carried out in this study were in accordance with relevant guidelines and regulations. Approval for this study (IRB#: 15-005140) was obtained from the Mayo Clinic IRB in accordance with the federal regulations and the principles of the Declaration of Helsinki.

### Study participants

We prospectively evaluated consecutive patients with MM and MGUS who underwent bone marrow aspirations as part of their standard of care clinical evaluation and who provided informed consent allowing the use of their bone marrow aspirate samples for this research project. The diagnostic criteria of International Myeloma Working Group (IMWG) were applied to confirm the diagnosis of MGUS and MM. Their bone marrow plasma was extracted from the bone marrow aspirate by centrifuging the aspirate sample at 2400 g for 10 minutes before then isolating the supernatant into a separate tube. The supernatant was then again centrifuged at 2400 g for 10 minutes before then isolating the plasma and storing it in EDTA tubes at −80 °C. The relevant laboratory data for the patients, including M-spike, serum free light chain (FLC) ratio, hemoglobin, total calcium, creatinine, β-2-microglobulin, Immunoglobulin subtype quantification, 24 hour urinary protein electrophoresis and immunofixation, BM clonal PC percentage and labelling index (S-phase %), cytogenetics and fluorescent *in situ* hybridization (FISH) results were abstracted for analysis.

### Bone marrow plasma sample preparation

The bone marrow plasma samples from patients were stored at −80 °C and shipped to Metabolon, Inc. (Research Triangle, NC) for batched analytic studies. Detailed procedure for the bone marrow plasma sample preparation is as previously described in a technical methods publication^24^ and summarized as follows. Samples were prepared using the automated MicroLab STAR system from Hamilton Company. Several recovery standards were added prior to the first step in the extraction process for QC purposes. To remove protein, dissociate small molecules bound to protein or trapped in the precipitated protein matrix, and to recover chemically diverse metabolites, proteins were precipitated with methanol under vigorous shaking for 2 min (Glen Mills GenoGrinder 2000) followed by centrifugation. Samples were placed briefly on a TurboVap (Zymark) to remove the organic solvent. The sample extracts were stored overnight under nitrogen before preparation for analysis.

#### Untargeted metabolomic analysis

Global untargeted metabolomic profiling was performed by Metabolon, Inc (Morrisville, NC) as described previously^24^ and summarized as follows. After extraction, samples were subjected to Ultrahigh Performance Liquid Chromatography-Tandem Mass Spectroscopy (UPLC-MS/MS) in the positive (two methods), negative (two methods) ion mode^25^. All methods utilized a Waters ACQUITY ultra-performance liquid chromatography (UPLC) and a Thermo Scientific Q-Exactive high resolution/accurate mass spectrometer interfaced with a heated electrospray ionization (HESI-II) source and Orbitrap mass analyzer operated at 35,000 mass resolution. The MS analysis alternated between MS and data-dependent MS^n^ scans using dynamic exclusion. The scan range varied slighted between methods but covered 70–1000 m/z.

#### Data extraction, compound identification and data normalization

Raw data were extracted, peak-identified and QC processed using Metabolon’s hardware and software as described previously^24^ and summarized as follows. Metabolites were identified by automated comparison of ion features to a reference library of chemical standards followed by visual inspection for quality control as previously described^26^. Peaks were quantified using area-under-the-curve. For studies spanning multiple days, a data normalization step was performed to correct variation resulting from instrument inter-day tuning differences. Essentially, each compound was corrected in run-day blocks by registering the medians to equal one (1.00) and normalizing each data point proportionately.

#### Metabolite set enrichment analysis (MSEA)

We performed MSEA tests to determine the metabolic pathways most different between the MGUS and MM groups using the overrepresentation analysis which was based on the enrichment of metabolites in a metabolic pathway compared to the total of annotated metabolites in the same pathway. Metaboanalyst 4.0 and its library of 99-metabolite sets of normal human metabolic pathways (SMPDB) was used to carry out this statistical analysis^27^.

#### Quantification of complex lipids

Complex lipid analysis was performed by Metabolon, Inc as previously described^28^ and summarized as follows. Lipids were extracted from samples in methanol:dichloromethane in a modified Bligh-Dyer extraction in the presence of internal standards^28^. The extracts were concentrated under nitrogen and reconstituted in 0.25 mL of 10 mM ammonium acetate dichloromethane:methanol (50:50) before being transferred to inserts and placed in vials for infusion-MS analysis, performed on a Shimadzu LC with nano PEEK tubing and the Sciex SelexIon-5500 QTRAP^28^. The samples were analyzed via both positive and negative mode electrospray and the 5500 QTRAP scan was performed in MRM mode with the total of more than 1,100 MRMs^28^. Individual lipid species were quantified based on single point quantification using 55 isotopically labeled internal standards^28^. Finally, lipid class concentrations were calculated from the sum of all molecular species within a class, and fatty acid compositions were determined by calculating the proportion of each class comprised by individual fatty acids^28^.

#### Quantification of AA and kynurenine analysis

Quantitative analysis of AAs and kynurenine metabolites were performed by the Mayo Clinic Metabolomics Core as previously described^29^ and summarized as follows. Plasma samples and amino acid calibration standards were prepared with MassTrak Amino Acid Analysis Solution (AAA) kit from Waters according to instructions with slight modifications for detection on a mass spectrometer^29^. A 10-point standard concentration curve was made from the calibration standard solution to calculate amino acid concentrations in plasma samples^29^. A solution containing 19 isotopes purchased from Cambridge Isotope Laboratories, Isotec and MassTrace was used as the internal standard solution^29^. Frozen plasma samples were thawed, spiked with internal standard then deproteinized with cold MeOH followed by centrifugation at 10,000 g for 5 minutes prior to derivatization with the derivatizing reagent 6-aminoquinolyl-N-hydroxysuccinimidyl carbamate according to MassTrak instructions^29^. High resolution separation was done using an Acquity UPLC system, injecting 1 µl of derivatized solution, with a UPLC BEH C18 1.7 micron 2.1 × 150 mm column from Waters^29^. Column flow was set to 400 µl/min with a gradient from 99.9%A to 98%B where buffer A is 1% acetonitrile in 0.1% formic acid and buffer B is 100% acetonitrile^29^. A column temp of 43 degrees Celsius and a sample tray temp of 6% Celsius was set and mass detection was completed on a TSQ Quantum Ultra from Thermo Finnigan running in positive ESI mode^29^. Finally, the settings of a scan width of 0.002, scan time of 0.04 seconds per transition mass, collision energy of 25, collision gas pressure of 1.5 mTorr, tube lens value set to 90 and monitoring a signature ion of the derivatized amines at m/z 171.04 by selected reaction monitoring were employed^29^. By using these aforementioned methods, a total of 42 amino acids and metabolites were able to be measured.

### Statistical analysis

For statistical analyses of the untargeted profiling and data display, any missing values were assumed to be below the limits of detection; these values will be imputed with the compound minimum (minimum value imputation). Statistical analysis of log-transformed metabolomic data was performed in ArrayStudio (Omicsoft) to compare data between experimental groups using ANOVA. For the post-hoc contrasts p-values and false discovery rate (FDR) were calculated according to method proposed by Storey and Tibshirani^30^. Resulting q-values were assessed across entire dataset and significance was defined as p ≤ 0.05 and q ≤ 0.10. The areas under the curve (AUC) of ROC curves were used to determine the diagnostic effectiveness of the top four metabolites and top four complex lipids responsible for group separation between MGUS and MM using Metaboanalyst 4.0^27^.

For the quantitative levels of metabolites and complex lipids between groups, given the non-normal distribution of the data, the differences between groups of interest were compared and analyzed using the non-parametric Mann-Whitney U test, and significance was defined as p < 0.05. This analysis was performed by using GraphPad Prism version 7.00 for Windows, GraphPad Software, La Jolla California USA, www.graphpad.com.

#### Hierarchical clustering analysis

Hierarchical clustering is an unsupervised method for clustering the data and can show large-scale differences. Hierarchical clustering was performed in ArrayStudio (Omicsoft) for untargeted profiling and complex lipid panel results. These analyses were done on log-transformed data utilizing the Euclidian distance metrics.

#### Random forest analysis

Random Forest analyses were conducted in the R software separately for untargeted metabolomic profiling and complex lipid panel datasets. Results for all samples and all detected metabolites were used for analysis. Random forest represents a supervised classification technique based on biochemical profile of the sample. To build the training set, half of the samples from each group are selected (i.e. “training set”), while the remaining half is used as the test set (i.e. “out-of-bag” (OOB) variables). The test set samples are passed down the tree to obtain a class prediction for each sample. The process of building the decision tree and testing it is repeated 50,000 times. The final classification of each sample is determined by computing the class prediction frequency (“votes”) for the OOB variables over the whole forest^31^.

## Supplementary information

Supplemental information.

Supplemental information2.

Supplemental information3.
