# Supplementary material for: Metabolomic and Lipidomic Profiling of Bone Marrow Plasma Differentiates Patients with Monoclonal Gammopathy of Undetermined Significance from Multiple Myeloma
Source: Sci Rep. 2020 Jun 24;10:10250. doi: 10.1038/s41598-020-67105-3 (PMC7314797; doi:10.1038/s41598-020-67105-3)
Supplement: Supplementary file 1 — Supplemental information. [file 41598_2020_67105_MOESM1_ESM.pdf]

Supplementary Information:

Metabolomic and Lipidomic Profiling of Bone Marrow Plasma Differentiates Patients with Monoclonal Gammopathy of Undetermined Significance from Multiple Myeloma

Wilson I. Gonsalves[1], Katarzyna Broniowska[5], Erik Jessen[2], Xuan-Mai Petterson[3], Alexander Graham Bush[3], Jaimee Gransee[3], Martha Q. Lacy[1], Taro Hitosugi[3] and Shaji K. Kumar[1]

From the Division of Hematology[1], Biostatistics and Informatics[2], Endocrinology[3] and the Department of Oncology, Molecular Therapeutics[4], Mayo Clinic, Rochester, MN. Metabolon Inc, Morrisville, NC[5].

Supplementary Table 1: Clinical characteristics of the 50 patients with either MGUS, NDMM or RRMM.

**Characteristics**	**MGUS (N = 25)**	**NDMM (N = 16)**	**RRMM (N = 9)**
Age	69 (41 – 89)	62 (42 – 86)	71 (44 – 77)
Male	19 (76%)	7 (44%)	6 (67%)
BMPC%	5 (0 – 9)	35 (5 – 90)	40 (10 – 80)
S-phase %	0.5 (0 – 2.8)	0.6 (0.4 – 8.4)	3.1 (0.4 – 6.6)
M-spike	0.8 (0 – 1.4)	2.5 (0.4 – 4.6)	2.3 (1.3 – 4.5)
M-protein Isotype			
IgG	12	5	1
IgA	6	7	4
IgM	3	0	0
Light chain only	4	4	4
Creatinine	1.1 (0.7 – 2.8)	0.9 (0.4 – 7.1)	1 (0.8 – 1.7)
Hemoglobin	13 (9.7 – 17.3)	12.2 (9.1 – 14.8)	11.4 (7.3 – 13.9)
Plasma cell FISH			
Hyperdiploid	8	8	5
t(4;14)	0	0	0
t(14;16)	0	1	2
t(11;14)	2	4	2
Other	4	3	0
BMI	28 (21 - 51)	29 (21 – 40)	26 (16 – 34)

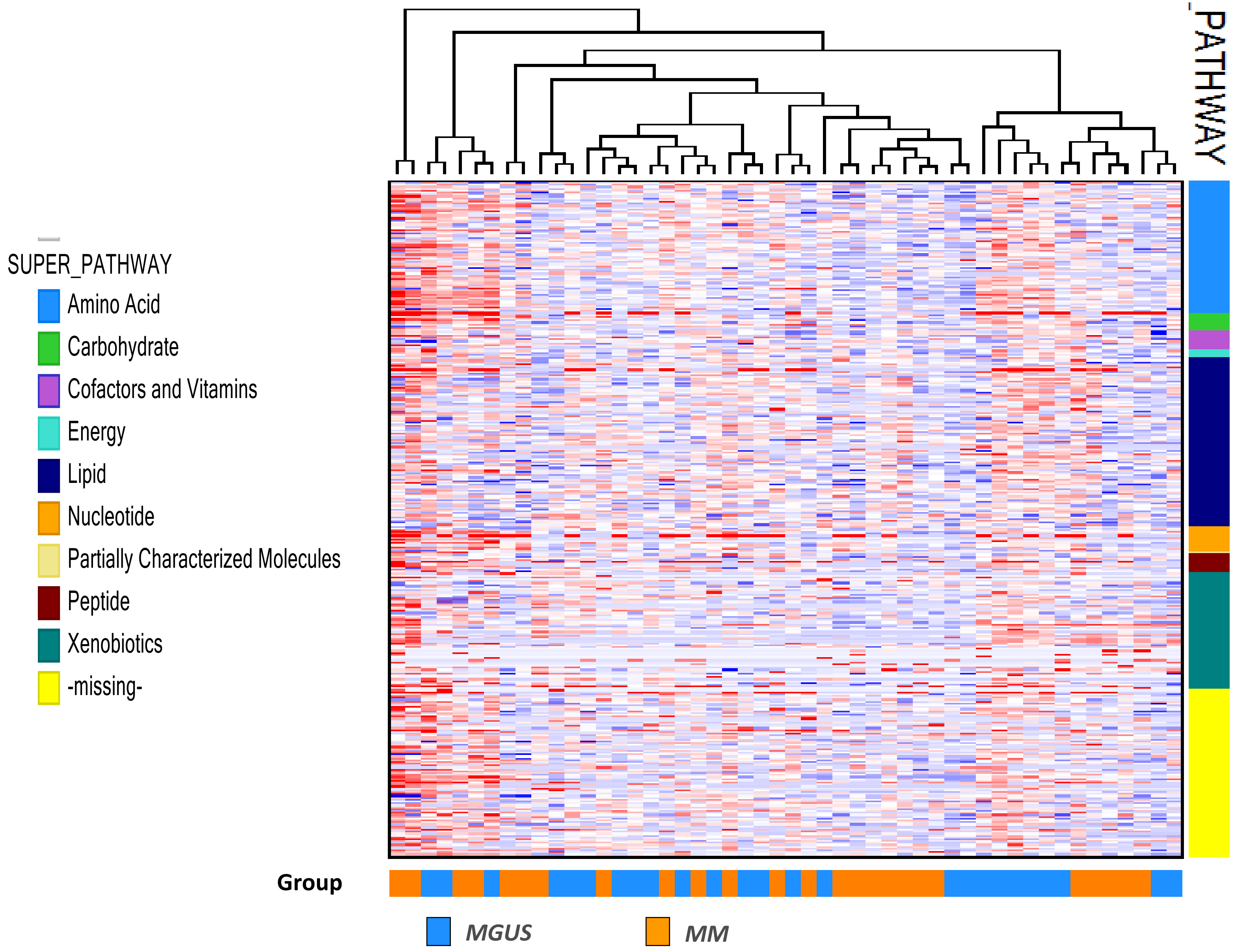

Supplementary Figure 1: Hierarchical clustering analysis of bone marrow plasma samples from patients with MGUS (N = 25) and MM (N =25) based on relative levels of metabolites.

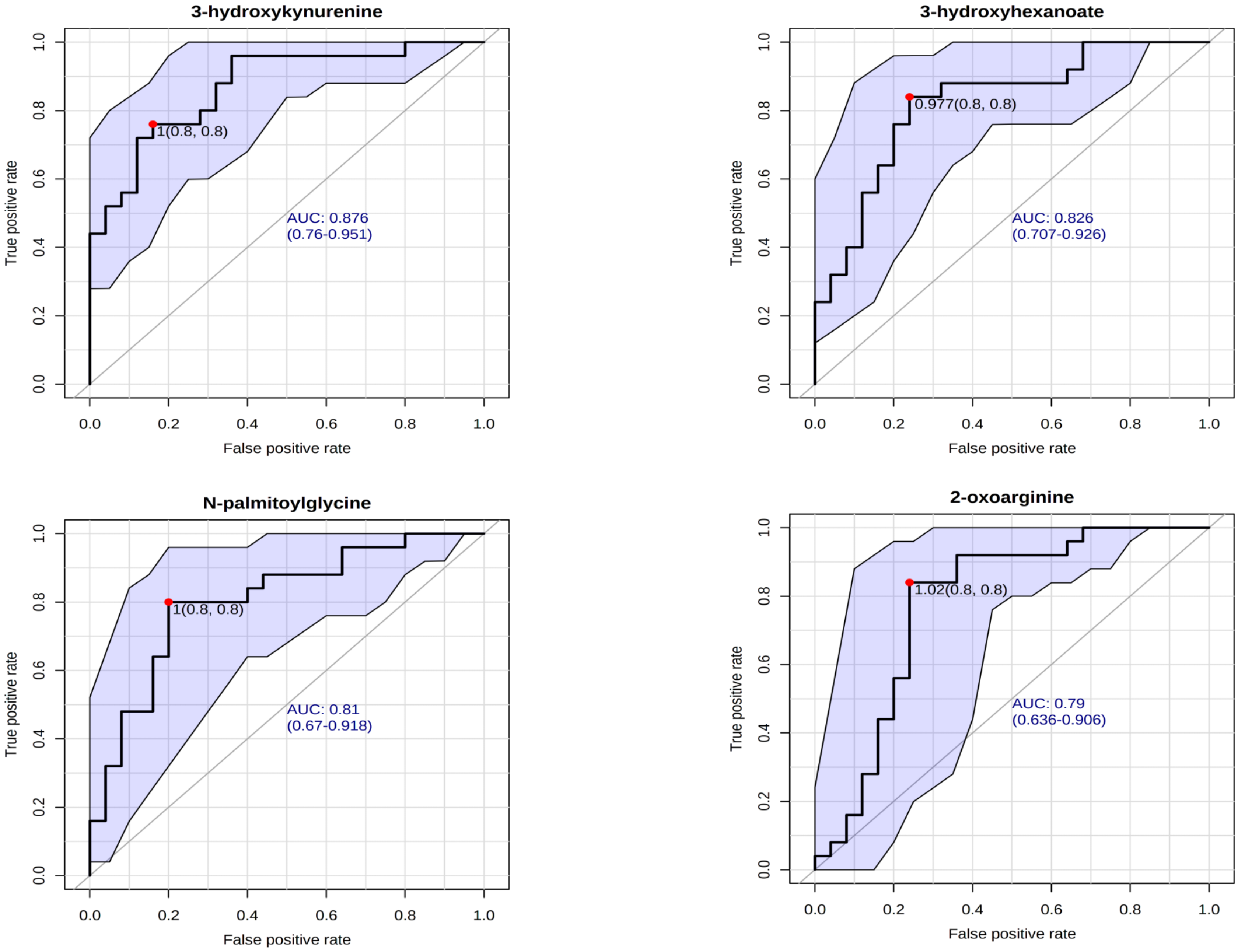

Supplementary Figure 2: Receiver operator characteristic analysis of the top four metabolites responsible for group separation between MGUS and MM.

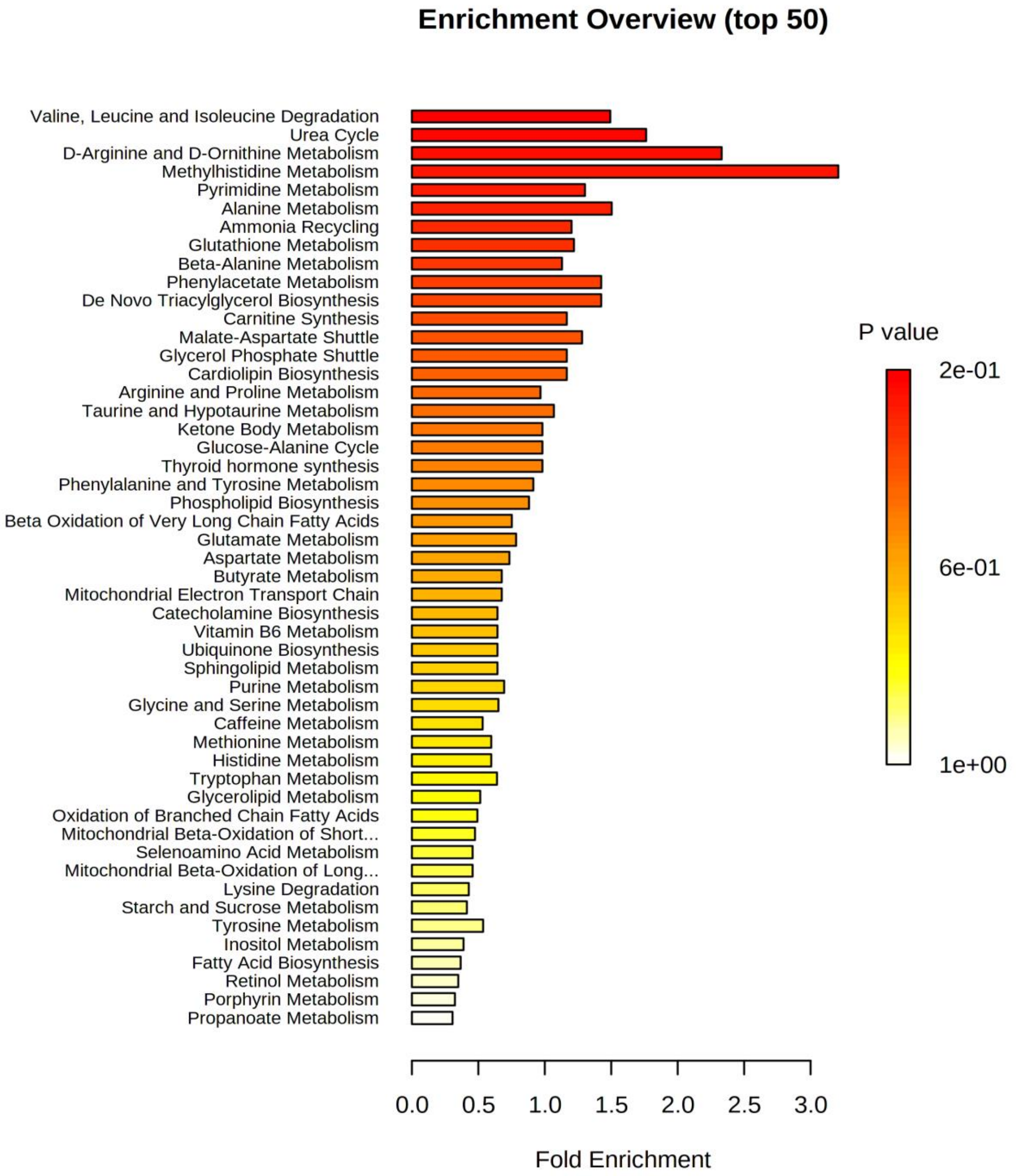

Supplementary Figure 3: Metabolite set enrichment analysis (MSEA) of MM compared to MGUS using MetaboAnalyst 4.0.

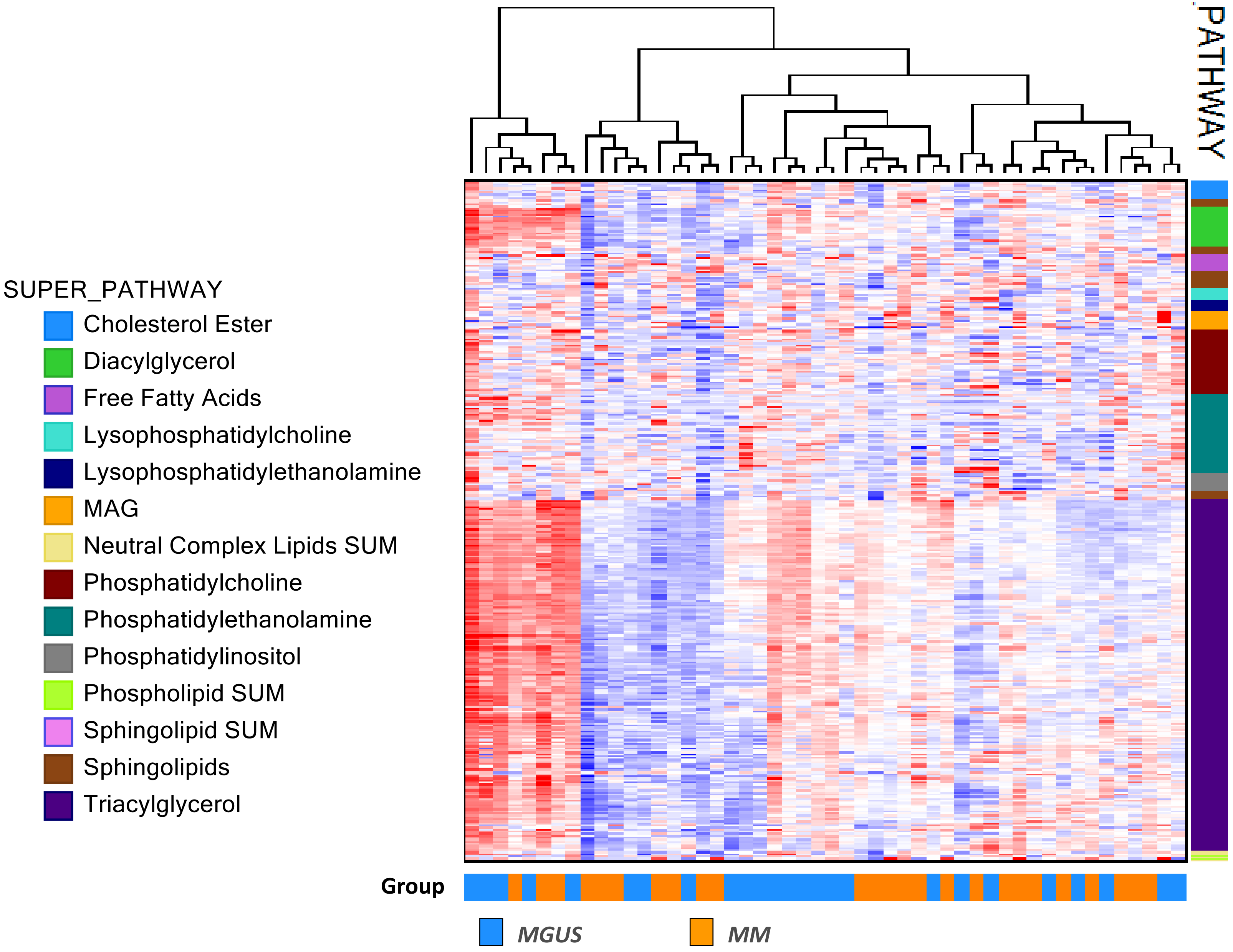

Supplementary Figure 4: Hierarchical clustering analysis of bone marrow plasma samples from patients with MGUS (N = 25) and MM (N =25) based on concentrations of complex lipids.

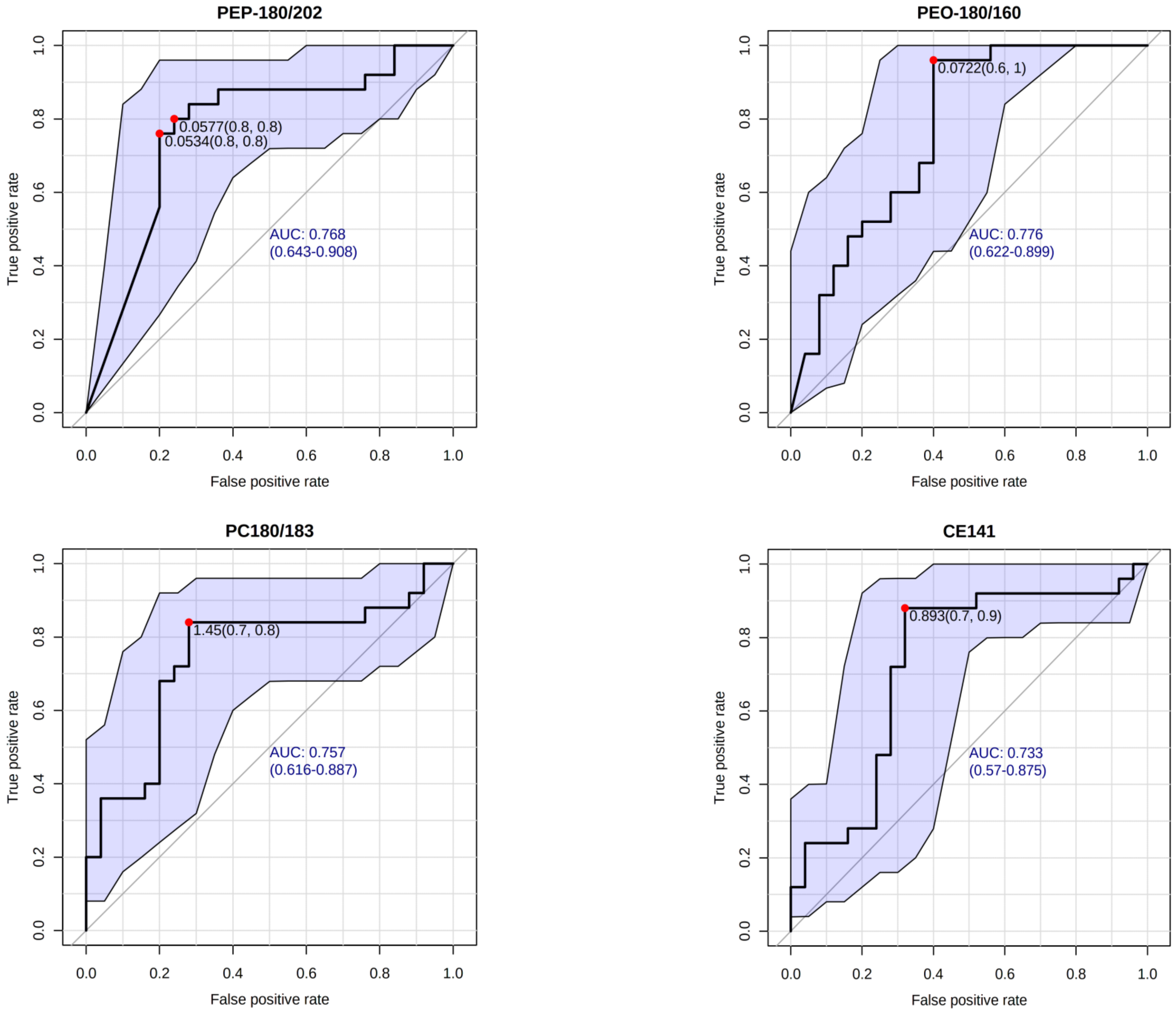

Supplementary Figure 5: Receiver operator characteristic analysis of the top four complex lipids responsible for group separation between MGUS and MM.
