# Supplementary material for: Metabolomic and Lipidomic Profiling of Bone Marrow Plasma Differentiates Patients with Monoclonal Gammopathy of Undetermined Significance from Multiple Myeloma
Source: Sci Rep. 2020 Jun 24;10:10250. doi: 10.1038/s41598-020-67105-3 (PMC7314797; doi:10.1038/s41598-020-67105-3)
Supplement: Supplementary file 2 — Supplemental information2. [file 41598_2020_67105_MOESM2_ESM.docx]

| **SUPPLEMENTARY TABLE 2: Heat map of statistically significant biochemicals profiled in this study. Red and green shaded cells indicate p≤0.05 (red indicates that the mean values are significantly higher for that comparison; green values significantly lower). Light red and light green shaded cells indicate 0.05<p<0.10 (light red indicates that the mean values trend higher for that comparison; light green values trend lower).** | | | | **Fold change** |  |
| --- | --- | --- | --- | --- | --- |
|  |  |  |  |  |  |
| **Pathway Sort Order** | **Super Pathway** | **Sub Pathway** | **Biochemical Name** | **MM/**  **MGUS** |  |
| 1 | Amino Acid | Glycine, Serine and Threonine Metabolism | glycine | **1.35** |  |
| 2 |  |  | N-acetylglycine | **1.36** |  |
| 4 |  |  | sarcosine | **0.79** |  |
| 5 |  |  | dimethylglycine | 0.75 |  |
| 6 |  |  | betaine | 0.98 |  |
| 9 |  |  | serine | 1.01 |  |
| 10 |  |  | N-acetylserine | 1.06 |  |
| 15 |  |  | 2-methylserine | 0.93 |  |
| 16 |  |  | threonine | **0.83** |  |
| 17 |  |  | N-acetylthreonine | 1.17 |  |
| 18 |  |  | allo-threonine | 0.96 |  |
| 28 |  | Alanine and Aspartate Metabolism | alanine | **0.83** |  |
| 30 |  |  | N-acetylalanine | **1.11** |  |
| 31 |  |  | N-methylalanine | 0.62 |  |
| 32 |  |  | N,N-dimethylalanine | 0.64 |  |
| 34 |  |  | N-carbamoylalanine | 1.53 |  |
| 35 |  |  | aspartate | **1.79** |  |
| 36 |  |  | N-acetylaspartate (NAA) | 0.92 |  |
| 39 |  |  | asparagine | 0.97 |  |
| 41 |  |  | hydroxyasparagine** | 1.14 |  |
| 42 |  | Glutamate Metabolism | glutamate | 1.13 |  |
| 43 |  |  | glutamine | 0.96 |  |
| 44 |  |  | alpha-ketoglutaramate* | 1.03 |  |
| 45 |  |  | N-acetylglutamate | 1.33 |  |
| 46 |  |  | N-acetylglutamine | 1.05 |  |
| 48 |  |  | 4-hydroxyglutamate | 0.95 |  |
| 50 |  |  | gamma-carboxyglutamate | **1.39** |  |
| 52 |  |  | pyroglutamine* | 1.04 |  |
| 53 |  |  | N-acetyl-aspartyl-glutamate (NAAG) | 1.32 |  |
| 54 |  |  | beta-citrylglutamate | **0.70** |  |
| 56 |  |  | carboxyethyl-GABA | **1.50** |  |
| 60 |  |  | S-1-pyrroline-5-carboxylate | 0.88 |  |
| 66 |  | Histidine Metabolism | histidine | **0.86** |  |
| 67 |  |  | 1-methylhistidine | 0.76 |  |
| 68 |  |  | 3-methylhistidine | 1.19 |  |
| 69 |  |  | N-acetylhistidine | 0.90 |  |
| 70 |  |  | N-acetyl-3-methylhistidine* | 1.52 |  |
| 71 |  |  | N-acetyl-1-methylhistidine* | 1.03 |  |
| 72 |  |  | hydantoin-5-propionate | **1.98** |  |
| 73 |  |  | trans-urocanate | 0.89 |  |
| 74 |  |  | cis-urocanate | 1.06 |  |
| 75 |  |  | imidazole propionate | 2.25 |  |
| 76 |  |  | formiminoglutamate | 0.75 |  |
| 77 |  |  | imidazole lactate | 1.05 |  |
| 80 |  |  | N-acetylcarnosine | 1.07 |  |
| 83 |  |  | 1-methylhistamine | **0.39** |  |
| 84 |  |  | 1-methyl-4-imidazoleacetate | 1.66 |  |
| 85 |  |  | 1-methyl-5-imidazoleacetate | 1.08 |  |
| 86 |  |  | 1-ribosyl-imidazoleacetate* | 1.37 |  |
| 87 |  |  | 4-imidazoleacetate | 0.94 |  |
| 95 |  | Lysine Metabolism | lysine | 0.95 |  |
| 96 |  |  | N2-acetyllysine | 0.87 |  |
| 97 |  |  | N6-acetyllysine | **0.79** |  |
| 99 |  |  | N2-acetyl,N6-methyllysine | 0.57 |  |
| 100 |  |  | N2-acetyl,N6,N6-dimethyllysine | 2.43 |  |
| 104 |  |  | N6-methyllysine | 0.83 |  |
| 105 |  |  | N6,N6-dimethyllysine | 1.21 |  |
| 106 |  |  | N6,N6,N6-trimethyllysine | 0.80 |  |
| 107 |  |  | hydroxy-N6,N6,N6-trimethyllysine* | 1.53 |  |
| 108 |  |  | 5-hydroxylysine | 0.70 |  |
| 109 |  |  | 5-(galactosylhydroxy)-L-lysine | 1.32 |  |
| 111 |  |  | 2-aminoadipate | **0.54** |  |
| 115 |  |  | glutarylcarnitine (C5-DC) | **0.63** |  |
| 119 |  |  | pipecolate | 0.86 |  |
| 120 |  |  | 6-oxopiperidine-2-carboxylate | 1.12 |  |
| 125 |  |  | N,N,N-trimethyl-5-aminovalerate | 0.90 |  |
| 126 |  |  | fructosyllysine | **0.87** |  |
| 127 |  | Phenylalanine Metabolism | phenylalanine | 0.94 |  |
| 128 |  |  | N-acetylphenylalanine | 1.31 |  |
| 129 |  |  | 1-carboxyethylphenylalanine | 0.85 |  |
| 132 |  |  | phenylpyruvate | **0.83** |  |
| 133 |  |  | phenyllactate (PLA) | 0.84 |  |
| 137 |  |  | phenylacetate | **0.62** |  |
| 141 |  |  | 4-hydroxyphenylacetate | **1.26** |  |
| 144 |  | Tyrosine Metabolism | tyrosine | **0.83** |  |
| 145 |  |  | N-acetyltyrosine | 1.11 |  |
| 155 |  |  | 4-hydroxyphenylpyruvate | 0.90 |  |
| 157 |  |  | 4-hydroxyphenylacetatoylcarnitine | 0.49 |  |
| 158 |  |  | 3-(4-hydroxyphenyl)lactate | 0.86 |  |
| 162 |  |  | phenol sulfate | 1.09 |  |
| 163 |  |  | phenol glucuronide | 4.78 |  |
| 165 |  |  | 4-methoxyphenol sulfate | 1.95 |  |
| 173 |  |  | vanillactate | **2.18** |  |
| 174 |  |  | vanillylmandelate (VMA) | 1.44 |  |
| 176 |  |  | 3-methoxytyrosine | **1.34** |  |
| 182 |  |  | homovanillate (HVA) | **1.34** |  |
| 191 |  |  | gentisate | **2.04** |  |
| 196 |  |  | dopamine 3-O-sulfate | 0.85 |  |
| 197 |  |  | p-cresol glucuronide* | 5.17 |  |
| 198 |  |  | tyramine O-sulfate | 0.53 |  |
| 200 |  |  | vanillic alcohol sulfate | **1.46** |  |
| 214 |  |  | thyroxine | 1.06 |  |
| 219 |  | Tryptophan Metabolism | tryptophan | **0.76** |  |
| 226 |  |  | C-glycosyltryptophan | 1.23 |  |
| 228 |  |  | tryptophan betaine | 0.71 |  |
| 230 |  |  | kynurenine | 0.82 |  |
| 234 |  |  | kynurenate | 1.62 |  |
| 236 |  |  | N-formylanthranilic acid | 0.91 |  |
| 237 |  |  | anthranilate | 1.03 |  |
| 239 |  |  | 3-hydroxykynurenine | **6.05** |  |
| 240 |  |  | xanthurenate | 1.06 |  |
| 241 |  |  | 8-methoxykynurenate | **8.50** |  |
| 244 |  |  | picolinate | 0.88 |  |
| 249 |  |  | serotonin | **0.36** |  |
| 252 |  |  | 5-hydroxyindoleacetate | 2.34 |  |
| 256 |  |  | indolelactate | 0.77 |  |
| 257 |  |  | indoleacetate | 1.26 |  |
| 260 |  |  | indolepropionate | 0.67 |  |
| 262 |  |  | indoleacetylglutamine | 4.62 |  |
| 268 |  |  | indoleacetoylcarnitine* | **0.66** |  |
| 271 |  |  | 3-indoxyl sulfate | 0.92 |  |
| 272 |  |  | indoxyl glucuronide | **6.25** |  |
| 276 |  |  | 6-bromotryptophan | 0.87 |  |
| 279 |  | Leucine, Isoleucine and Valine Metabolism | leucine | **0.82** |  |
| 280 |  |  | N-acetylleucine | 0.93 |  |
| 284 |  |  | 4-methyl-2-oxopentanoate | **0.76** |  |
| 287 |  |  | alpha-hydroxyisocaproate | **0.75** |  |
| 288 |  |  | 2-ketocaprylate | 1.00 |  |
| 291 |  |  | isovalerate (i5:0) | 0.79 |  |
| 292 |  |  | isovalerylglycine | 1.25 |  |
| 293 |  |  | isovalerylcarnitine (C5) | **0.69** |  |
| 299 |  |  | 3-methylcrotonylglycine | **2.34** |  |
| 300 |  |  | beta-hydroxyisovalerate | 0.84 |  |
| 303 |  |  | 3-methylglutaconate | 1.25 |  |
| 307 |  |  | 3-methylglutarylcarnitine (2) | 1.58 |  |
| 316 |  |  | isoleucine | **0.87** |  |
| 318 |  |  | N-acetylisoleucine | 0.85 |  |
| 321 |  |  | 3-methyl-2-oxovalerate | **0.78** |  |
| 322 |  |  | 2-hydroxy-3-methylvalerate | 0.81 |  |
| 324 |  |  | 2-methylbutyrylcarnitine (C5) | **0.65** |  |
| 326 |  |  | 2-methylbutyrylglycine | 0.74 |  |
| 327 |  |  | tiglylcarnitine (C5:1-DC) | **2.15** |  |
| 328 |  |  | tigloylglycine | **1.86** |  |
| 330 |  |  | 3-hydroxy-2-ethylpropionate | 0.90 |  |
| 332 |  |  | ethylmalonate | **1.42** |  |
| 333 |  |  | methylsuccinate | **2.71** |  |
| 334 |  |  | methylsuccinoylcarnitine | 1.27 |  |
| 339 |  |  | valine | **0.78** |  |
| 340 |  |  | N-acetylvaline | 0.94 |  |
| 343 |  |  | 1-carboxyethylvaline | 0.65 |  |
| 345 |  |  | 3-methyl-2-oxobutyrate | **0.82** |  |
| 346 |  |  | alpha-hydroxyisovalerate | **0.79** |  |
| 348 |  |  | isobutyrylcarnitine (C4) | **0.53** |  |
| 349 |  |  | isobutyrylglycine | 0.77 |  |
| 350 |  |  | 3-hydroxyisobutyrate | 0.98 |  |
| 351 |  |  | 2,3-dihydroxy-2-methylbutyrate | 0.87 |  |
| 355 |  | Methionine, Cysteine, SAM and Taurine Metabolism | methionine | 0.89 |  |
| 356 |  |  | N-acetylmethionine | **1.22** |  |
| 357 |  |  | N-formylmethionine | **1.38** |  |
| 359 |  |  | methionine sulfone | 1.26 |  |
| 360 |  |  | methionine sulfoxide | **0.84** |  |
| 367 |  |  | 5-methylthioribose** | **1.19** |  |
| 368 |  |  | 2,3-dihydroxy-5-methylthio-4-pentenoate (DMTPA)* | 1.23 |  |
| 372 |  |  | cystathionine | 2.98 |  |
| 373 |  |  | alpha-ketobutyrate | 1.00 |  |
| 374 |  |  | cysteine | 0.90 |  |
| 376 |  |  | S-methylcysteine | 1.25 |  |
| 377 |  |  | S-methylcysteine sulfoxide | 1.32 |  |
| 379 |  |  | cysteine s-sulfate | **0.80** |  |
| 380 |  |  | cystine | **0.83** |  |
| 384 |  |  | lanthionine | 1.17 |  |
| 386 |  |  | cysteine sulfinic acid | 1.03 |  |
| 388 |  |  | hypotaurine | **0.70** |  |
| 389 |  |  | taurine | 0.92 |  |
| 390 |  |  | N-acetyltaurine | **1.28** |  |
| 391 |  |  | N-methyltaurine | 0.93 |  |
| 396 |  | Urea cycle; Arginine and Proline Metabolism | arginine | 0.96 |  |
| 398 |  |  | urea | **0.69** |  |
| 400 |  |  | ornithine | 0.99 |  |
| 402 |  |  | 3-amino-2-piperidone | 1.09 |  |
| 404 |  |  | 2-oxoarginine* | **0.44** |  |
| 405 |  |  | citrulline | **0.81** |  |
| 406 |  |  | homoarginine | **0.68** |  |
| 407 |  |  | homocitrulline | 0.96 |  |
| 408 |  |  | proline | 0.89 |  |
| 411 |  |  | dimethylarginine (SDMA + ADMA) | **1.13** |  |
| 412 |  |  | N-acetylarginine | **0.55** |  |
| 416 |  |  | N-acetylcitrulline | 0.54 |  |
| 417 |  |  | N-acetylproline | **4.35** |  |
| 418 |  |  | N-delta-acetylornithine | 0.76 |  |
| 419 |  |  | N-alpha-acetylornithine | 1.15 |  |
| 420 |  |  | N2,N5-diacetylornithine | 0.86 |  |
| 422 |  |  | N-methylhydroxyproline** | 0.36 |  |
| 423 |  |  | trans-4-hydroxyproline | 0.81 |  |
| 425 |  |  | pro-hydroxy-pro | 1.27 |  |
| 428 |  |  | N-methylproline | 0.60 |  |
| 429 |  |  | N,N,N-trimethyl-alanylproline betaine (TMAP) | 1.14 |  |
| 431 |  |  | N-monomethylarginine | 0.92 |  |
| 432 |  |  | argininate* | **0.37** |  |
| 436 |  |  | dimethylguanidino valeric acid (DMGV)* | 1.18 |  |
| 437 |  | Creatine Metabolism | guanidinoacetate | 1.03 |  |
| 438 |  |  | creatine | 0.88 |  |
| 439 |  |  | creatinine | 1.00 |  |
| 447 |  | Polyamine Metabolism | N-acetyl-isoputreanine* | **1.59** |  |
| 448 |  |  | spermidine | 1.65 |  |
| 450 |  |  | N('1)-acetylspermidine | 0.84 |  |
| 452 |  |  | acisoga | 1.26 |  |
| 455 |  |  | N1,N12-diacetylspermine | 0.56 |  |
| 456 |  |  | 5-methylthioadenosine (MTA) | **1.13** |  |
| 457 |  |  | N-acetylputrescine | 1.07 |  |
| 461 |  |  | 4-acetamidobutanoate | 1.20 |  |
| 465 |  | Guanidino and Acetamido Metabolism | 1-methylguanidine | 4.53 |  |
| 466 |  |  | 4-guanidinobutanoate | 0.79 |  |
| 467 |  |  | guanidinosuccinate | 0.77 |  |
| 473 |  | Glutathione Metabolism | cysteine-glutathione disulfide | **0.86** |  |
| 476 |  |  | cysteinylglycine | 1.00 |  |
| 477 |  |  | cysteinylglycine disulfide* | **0.81** |  |
| 478 |  |  | cys-gly, oxidized | 0.82 |  |
| 479 |  |  | 5-oxoproline | 1.32 |  |
| 480 |  |  | 2-aminobutyrate | 0.91 |  |
| 482 |  |  | 2-hydroxybutyrate/2-hydroxyisobutyrate | **0.87** |  |
| 528 | Peptide | Gamma-glutamyl Amino Acid | gamma-glutamylalanine | 0.89 |  |
| 530 |  |  | gamma-glutamylglutamate | 1.03 |  |
| 531 |  |  | gamma-glutamylglutamine | **0.89** |  |
| 532 |  |  | gamma-glutamylglycine | **1.52** |  |
| 533 |  |  | gamma-glutamylhistidine | 0.92 |  |
| 534 |  |  | gamma-glutamylisoleucine* | 0.80 |  |
| 535 |  |  | gamma-glutamylleucine | **0.82** |  |
| 536 |  |  | gamma-glutamyl-alpha-lysine | 0.95 |  |
| 537 |  |  | gamma-glutamyl-epsilon-lysine | 1.10 |  |
| 538 |  |  | gamma-glutamylmethionine | 0.85 |  |
| 539 |  |  | gamma-glutamylphenylalanine | 0.98 |  |
| 540 |  |  | gamma-glutamylthreonine | 0.88 |  |
| 542 |  |  | gamma-glutamyltyrosine | 0.87 |  |
| 543 |  |  | gamma-glutamylvaline | 0.78 |  |
| 545 |  |  | gamma-glutamylcitrulline* | **0.68** |  |
| 636 |  | Dipeptide | cyclo(gly-pro) | 1.65 |  |
| 757 |  |  | leucylalanine | 1.09 |  |
| 816 |  |  | phenylalanylglycine | 1.23 |  |
| 836 |  |  | prolylglycine | 0.98 |  |
| 919 |  |  | valylglycine | 1.19 |  |
| 986 |  | Fibrinogen Cleavage Peptide | Fibrinopeptide A* | 0.52 |  |
| 994 |  |  | Fibrinopeptide A, des-ala(1)* | 0.40 |  |
| 995 |  |  | Fibrinopeptide A, phosphono-ser(3)* | 0.29 |  |
| 1009 |  |  | Fibrinopeptide B (1-13)** | 0.39 |  |
| 1011 |  | Acetylated Peptides | phenylacetylcarnitine | **0.38** |  |
| 1013 |  |  | phenylacetylglutamate | 5.09 |  |
| 1014 |  |  | phenylacetylglutamine | 1.85 |  |
| 1016 |  |  | 4-hydroxyphenylacetylglutamine | 3.44 |  |
| 1046 | Carbohydrate | Glycolysis, Gluconeogenesis, and Pyruvate Metabolism | 1,5-anhydroglucitol (1,5-AG) | **0.77** |  |
| 1049 |  |  | glucose | 0.95 |  |
| 1063 |  |  | 3-phosphoglycerate | 1.18 |  |
| 1065 |  |  | pyruvate | 1.04 |  |
| 1066 |  |  | lactate | **0.93** |  |
| 1069 |  |  | glycerate | **0.91** |  |
| 1088 |  | Pentose Metabolism | ribitol | 1.07 |  |
| 1089 |  |  | ribonate | **1.37** |  |
| 1093 |  |  | xylose | 0.76 |  |
| 1095 |  |  | arabinose | 1.05 |  |
| 1103 |  |  | arabitol/xylitol | 1.32 |  |
| 1109 |  |  | arabonate/xylonate | 1.12 |  |
| 1112 |  |  | sedoheptulose | 1.16 |  |
| 1113 |  |  | ribulonate/xylulonate/lyxonate* | 0.87 |  |
| 1122 |  | Glycogen Metabolism | maltose | 0.80 |  |
| 1127 |  | Disaccharides and Oligosaccharides | lactose | 1.21 |  |
| 1146 |  |  | sucrose | 3.74 |  |
| 1165 |  | Fructose, Mannose and Galactose Metabolism | fructose | 1.23 |  |
| 1170 |  |  | mannitol/sorbitol | 0.75 |  |
| 1171 |  |  | mannose | 1.02 |  |
| 1191 |  |  | galactonate | 0.91 |  |
| 1223 |  | Aminosugar Metabolism | glucuronate | **1.75** |  |
| 1234 |  |  | N-acetylneuraminate | 0.98 |  |
| 1245 |  |  | N-acetylglucosaminylasparagine | **0.66** |  |
| 1246 |  |  | erythronate* | **1.11** |  |
| 1248 |  |  | N-acetylglucosamine/N-acetylgalactosamine | **1.33** |  |
| 1254 | Energy | TCA Cycle | citrate | 0.97 |  |
| 1256 |  |  | aconitate [cis or trans] | 1.08 |  |
| 1258 |  |  | isocitrate | 0.94 |  |
| 1259 |  |  | isocitric lactone | 1.02 |  |
| 1260 |  |  | alpha-ketoglutarate | 0.90 |  |
| 1262 |  |  | succinylcarnitine (C4-DC) | 0.91 |  |
| 1263 |  |  | succinate | 0.99 |  |
| 1264 |  |  | fumarate | 0.97 |  |
| 1266 |  |  | malate | 0.99 |  |
| 1275 |  |  | citraconate/glutaconate | 1.24 |  |
| 1276 |  |  | 2-methylcitrate/homocitrate | 1.08 |  |
| 1280 |  | Oxidative Phosphorylation | phosphate | 1.02 |  |
| 1282 | Lipid | Fatty Acid Synthesis | malonylcarnitine | 0.84 |  |
| 1284 |  |  | 2-methylmalonylcarnitine (C4-DC) | 0.94 |  |
| 1298 |  | Short Chain Fatty Acid | pentoic acid* | 0.89 |  |
| 1299 |  | Medium Chain Fatty Acid | caproate (6:0) | 0.89 |  |
| 1301 |  |  | caprylate (8:0) | 1.04 |  |
| 1303 |  |  | caprate (10:0) | 0.96 |  |
| 1304 |  |  | cis-4-decenoate (10:1n6)* | 0.99 |  |
| 1305 |  |  | undecanoate (11:0) | 0.90 |  |
| 1306 |  |  | 10-undecenoate (11:1n1) | 1.14 |  |
| 1307 |  |  | laurate (12:0) | 1.15 |  |
| 1308 |  |  | 5-dodecenoate (12:1n7) | 1.09 |  |
| 1310 |  | Long Chain Saturated Fatty Acid | myristate (14:0) | 1.01 |  |
| 1311 |  |  | pentadecanoate (15:0) | 0.99 |  |
| 1312 |  |  | palmitate (16:0) | 1.05 |  |
| 1313 |  |  | margarate (17:0) | 1.00 |  |
| 1314 |  |  | stearate (18:0) | 1.04 |  |
| 1315 |  |  | nonadecanoate (19:0) | 1.08 |  |
| 1316 |  |  | arachidate (20:0) | 1.06 |  |
| 1323 |  | Long Chain Monounsaturated Fatty Acid | myristoleate (14:1n5) | 1.05 |  |
| 1326 |  |  | palmitoleate (16:1n7) | 1.11 |  |
| 1328 |  |  | 10-heptadecenoate (17:1n7) | 1.00 |  |
| 1330 |  |  | oleate/vaccenate (18:1) | 1.10 |  |
| 1336 |  |  | 10-nonadecenoate (19:1n9) | 1.10 |  |
| 1339 |  |  | eicosenoate (20:1) | 1.12 |  |
| 1343 |  |  | erucate (22:1n9) | 0.92 |  |
| 1346 |  | Long Chain Polyunsaturated Fatty Acid (n3 and n6) | tetradecadienoate (14:2)* | 1.18 |  |
| 1353 |  |  | stearidonate (18:4n3) | 0.87 |  |
| 1356 |  |  | eicosapentaenoate (EPA; 20:5n3) | 0.92 |  |
| 1358 |  |  | docosapentaenoate (n3 DPA; 22:5n3) | 1.03 |  |
| 1359 |  |  | docosahexaenoate (DHA; 22:6n3) | 1.05 |  |
| 1360 |  |  | docosatrienoate (22:3n3) | 1.01 |  |
| 1361 |  |  | nisinate (24:6n3) | **0.68** |  |
| 1362 |  |  | hexadecadienoate (16:2n6) | 1.08 |  |
| 1363 |  |  | linoleate (18:2n6) | 1.11 |  |
| 1365 |  |  | linolenate [alpha or gamma; (18:3n3 or 6)] | 1.08 |  |
| 1366 |  |  | dihomo-linoleate (20:2n6) | 1.08 |  |
| 1368 |  |  | dihomo-linolenate (20:3n3 or n6) | 0.98 |  |
| 1369 |  |  | arachidonate (20:4n6) | 1.06 |  |
| 1370 |  |  | docosatrienoate (22:3n6)* | 0.71 |  |
| 1371 |  |  | adrenate (22:4n6) | 0.98 |  |
| 1372 |  |  | docosapentaenoate (n6 DPA; 22:5n6) | 0.94 |  |
| 1373 |  |  | docosadienoate (22:2n6) | 1.00 |  |
| 1375 |  |  | mead acid (20:3n9) | 0.86 |  |
| 1428 |  | Fatty Acid, Branched | (12 or 13)-methylmyristate (a15:0 or i15:0) | 0.96 |  |
| 1431 |  |  | (14 or 15)-methylpalmitate (a17:0 or i17:0) | 1.01 |  |
| 1435 |  |  | (16 or 17)-methylstearate (a19:0 or i19:0) | 1.00 |  |
| 1452 |  | Fatty Acid, Dicarboxylate | glutarate (C5-DC) | **1.23** |  |
| 1455 |  |  | 3-methylglutarate/2-methylglutarate | **2.13** |  |
| 1456 |  |  | 2-hydroxyglutarate | **1.63** |  |
| 1457 |  |  | 4-hydroxy-2-oxoglutaric acid | 0.82 |  |
| 1458 |  |  | adipate (C6-DC) | 0.46 |  |
| 1460 |  |  | 2-hydroxyadipate | 1.40 |  |
| 1461 |  |  | 3-hydroxyadipate* | **2.49** |  |
| 1462 |  |  | 3-methyladipate | 1.92 |  |
| 1464 |  |  | maleate | 1.20 |  |
| 1465 |  |  | pimelate (C7-DC) | 1.97 |  |
| 1466 |  |  | heptenedioate (C7:1-DC)* | **1.88** |  |
| 1468 |  |  | suberate (C8-DC) | **2.01** |  |
| 1471 |  |  | azelate (C9-DC) | **1.21** |  |
| 1472 |  |  | nonenedioate (C9:1-DC)* | **3.27** |  |
| 1473 |  |  | sebacate (C10-DC) | **1.91** |  |
| 1475 |  |  | 2-hydroxysebacate | **2.21** |  |
| 1476 |  |  | dodecadienoate (12:2)* | 1.01 |  |
| 1478 |  |  | dodecanedioate (C12-DC) | **1.71** |  |
| 1479 |  |  | 3-hydroxydodecanedioate* | **2.09** |  |
| 1480 |  |  | tridecenedioate (C13:1-DC)* | **1.42** |  |
| 1481 |  |  | tetradecanedioate (C14-DC) | 1.35 |  |
| 1483 |  |  | branched chain 14:0 dicarboxylic acid** | 0.56 |  |
| 1484 |  |  | hexadecanedioate (C16-DC) | 1.01 |  |
| 1486 |  |  | hexadecenedioate (C16:1-DC)* | 1.11 |  |
| 1488 |  |  | octadecanedioate (C18-DC) | 0.86 |  |
| 1489 |  |  | octadecenedioate (C18:1-DC)* | 0.96 |  |
| 1490 |  |  | tetradecadienedioate (C14:2-DC)* | **1.59** |  |
| 1491 |  |  | octadecadienedioate (C18:2-DC)* | 0.72 |  |
| 1493 |  |  | eicosanedioate (C20-DC) | 0.86 |  |
| 1494 |  |  | eicosenedioate (C20:1-DC)* | 0.94 |  |
| 1495 |  |  | docosadioate (C22-DC) | **0.69** |  |
| 1496 |  |  | 3-carboxy-4-methyl-5-propyl-2-furanpropanoate (CMPF) | 0.35 |  |
| 1497 |  |  | hydroxy-CMPF* | 0.56 |  |
| 1498 |  |  | 3-carboxy-4-methyl-5-pentyl-2-furanpropionate (3-CMPFP)** | 0.72 |  |
| 1540 |  | Fatty Acid, Amino | 2-aminoheptanoate | 1.00 |  |
| 1545 |  |  | 2-aminooctanoate | 1.18 |  |
| 1546 |  |  | N-acetyl-2-aminooctanoate* | 0.95 |  |
| 1558 |  | Fatty Acid Metabolism (also BCAA Metabolism) | butyrylcarnitine (C4) | 0.90 |  |
| 1559 |  |  | butyrylglycine | 0.91 |  |
| 1561 |  |  | propionylcarnitine (C3) | **0.57** |  |
| 1562 |  |  | propionylglycine | 0.80 |  |
| 1564 |  |  | methylmalonate (MMA) | **1.26** |  |
| 1565 |  | Fatty Acid Metabolism (Acyl Glutamine) | hexanoylglutamine | **1.84** |  |
| 1577 |  | Fatty Acid Metabolism (Acyl Glycine) | hexanoylglycine | 1.03 |  |
| 1583 |  |  | N-palmitoylglycine | **1.52** |  |
| 1590 |  |  | 3-hydroxybutyroylglycine** | **2.68** |  |
| 1593 |  |  | picolinoylglycine | 2.57 |  |
| 1594 |  | Fatty Acid Metabolism (Acyl Carnitine, Short Chain) | acetylcarnitine (C2) | 0.99 |  |
| 1597 |  | Fatty Acid Metabolism (Acyl Carnitine, Medium Chain) | hexanoylcarnitine (C6) | **0.81** |  |
| 1598 |  |  | octanoylcarnitine (C8) | **0.76** |  |
| 1599 |  |  | nonanoylcarnitine (C9) | **0.66** |  |
| 1600 |  |  | decanoylcarnitine (C10) | **0.77** |  |
| 1601 |  |  | laurylcarnitine (C12) | 0.83 |  |
| 1602 |  | Fatty Acid Metabolism (Acyl Carnitine, Long Chain Saturated) | myristoylcarnitine (C14) | 0.92 |  |
| 1604 |  |  | palmitoylcarnitine (C16) | 0.99 |  |
| 1605 |  |  | margaroylcarnitine (C17)* | **0.88** |  |
| 1606 |  |  | stearoylcarnitine (C18) | 1.00 |  |
| 1607 |  |  | arachidoylcarnitine (C20)* | 1.07 |  |
| 1608 |  |  | behenoylcarnitine (C22)* | 0.87 |  |
| 1609 |  |  | lignoceroylcarnitine (C24)* | **0.77** |  |
| 1610 |  |  | cerotoylcarnitine (C26)* | **0.71** |  |
| 1616 |  | Fatty Acid Metabolism (Acyl Carnitine, Monounsaturated) | 3-decenoylcarnitine | **0.75** |  |
| 1617 |  |  | cis-4-decenoylcarnitine (C10:1) | 0.80 |  |
| 1618 |  |  | 5-dodecenoylcarnitine (C12:1) | 0.84 |  |
| 1620 |  |  | myristoleoylcarnitine (C14:1)* | 0.92 |  |
| 1621 |  |  | palmitoleoylcarnitine (C16:1)* | 0.99 |  |
| 1622 |  |  | oleoylcarnitine (C18:1) | 1.05 |  |
| 1623 |  |  | eicosenoylcarnitine (C20:1)* | 1.05 |  |
| 1624 |  |  | erucoylcarnitine (C22:1)* | 0.88 |  |
| 1625 |  |  | nervonoylcarnitine (C24:1)* | **0.80** |  |
| 1626 |  |  | ximenoylcarnitine (C26:1)* | **0.75** |  |
| 1627 |  | Fatty Acid Metabolism (Acyl Carnitine, Polyunsaturated) | linoleoylcarnitine (C18:2)* | 0.98 |  |
| 1628 |  |  | linolenoylcarnitine (C18:3)* | 0.90 |  |
| 1629 |  |  | dihomo-linoleoylcarnitine (C20:2)* | 0.98 |  |
| 1630 |  |  | arachidonoylcarnitine (C20:4) | 0.81 |  |
| 1631 |  |  | dihomo-linolenoylcarnitine (C20:3n3 or 6)* | 0.86 |  |
| 1635 |  |  | adrenoylcarnitine (C22:4)* | 0.99 |  |
| 1636 |  |  | docosapentaenoylcarnitine (C22:5n3)* | **0.78** |  |
| 1640 |  | Fatty Acid Metabolism (Acyl Carnitine, Dicarboxylate) | adipoylcarnitine (C6-DC) | 0.88 |  |
| 1645 |  |  | pimeloylcarnitine/3-methyladipoylcarnitine (C7-DC) | **1.54** |  |
| 1646 |  |  | suberoylcarnitine (C8-DC) | 1.23 |  |
| 1648 |  |  | octadecanedioylcarnitine (C18-DC)* | 0.93 |  |
| 1649 |  |  | octadecenedioylcarnitine (C18:1-DC)* | 1.06 |  |
| 1650 |  | Fatty Acid Metabolism (Acyl Carnitine, Hydroxy) | (R)-3-hydroxybutyrylcarnitine | 1.46 |  |
| 1651 |  |  | (S)-3-hydroxybutyrylcarnitine | 1.19 |  |
| 1655 |  |  | 3-hydroxydecanoylcarnitine | **0.81** |  |
| 1657 |  |  | 3-hydroxyoleoylcarnitine | 1.03 |  |
| 1658 |  | Carnitine Metabolism | deoxycarnitine | 0.95 |  |
| 1659 |  |  | carnitine | **0.84** |  |
| 1662 |  | Ketone Bodies | acetoacetate | **2.22** |  |
| 1664 |  |  | 3-hydroxybutyrate (BHBA) | **3.10** |  |
| 1668 |  | Fatty Acid Metabolism (Acyl Choline) | palmitoylcholine | 1.72 |  |
| 1669 |  |  | oleoylcholine | 1.43 |  |
| 1672 |  |  | dihomo-linolenoyl-choline | 1.17 |  |
| 1673 |  |  | linoleoylcholine* | 1.40 |  |
| 1675 |  |  | stearoylcholine* | **1.37** |  |
| 1676 |  |  | docosahexaenoylcholine | **1.11** |  |
| 1677 |  |  | arachidonoylcholine | **1.45** |  |
| 1685 |  | Fatty Acid, Monohydroxy | 2-hydroxyoctanoate | 1.07 |  |
| 1686 |  |  | 2-hydroxydecanoate | 0.91 |  |
| 1687 |  |  | 2-hydroxylaurate | 0.85 |  |
| 1689 |  |  | 2-hydroxypalmitate | **1.27** |  |
| 1690 |  |  | 2-hydroxyoleate | 1.03 |  |
| 1691 |  |  | 2-hydroxystearate | **1.11** |  |
| 1693 |  |  | 2-hydroxybehenate | 0.89 |  |
| 1695 |  |  | 2-hydroxynervonate* | 1.07 |  |
| 1698 |  |  | 3-hydroxyhexanoate | **1.84** |  |
| 1699 |  |  | 3-hydroxyoctanoate | **1.53** |  |
| 1701 |  |  | 3-hydroxydecanoate | **1.25** |  |
| 1702 |  |  | 3-hydroxysebacate | **2.87** |  |
| 1703 |  |  | 3-hydroxylaurate | 1.15 |  |
| 1704 |  |  | 3-hydroxymyristate | 1.16 |  |
| 1705 |  |  | 3-hydroxypalmitate | 1.06 |  |
| 1706 |  |  | 3-hydroxyoleate* | 1.12 |  |
| 1714 |  |  | 7-hydroxyoctanoate | 1.11 |  |
| 1717 |  |  | 16-hydroxypalmitate | 0.84 |  |
| 1720 |  |  | 13-HODE + 9-HODE | 1.13 |  |
| 1734 |  |  | 3-hydroxystearate | **0.86** |  |
| 1735 |  |  | 9-hydroxystearate | 0.93 |  |
| 1743 |  | Fatty Acid, Dihydroxy | 12,13-DiHOME | 1.08 |  |
| 1744 |  |  | 9,10-DiHOME | 1.03 |  |
| 1745 |  |  | 2S,3R-dihydroxybutyrate | 0.92 |  |
| 1746 |  |  | 2R,3R-dihydroxybutyrate | **0.67** |  |
| 1749 |  |  | 3,4-dihydroxybutyrate | **1.15** |  |
| 1834 |  | Eicosanoid | 12-HETE | 0.57 |  |
| 1848 |  | Endocannabinoid | oleoyl ethanolamide | 1.27 |  |
| 1851 |  |  | palmitoyl ethanolamide | 1.02 |  |
| 1852 |  |  | stearoyl ethanolamide | 1.03 |  |
| 1856 |  |  | arachidonoyl ethanolamide | 1.13 |  |
| 1861 |  |  | N-oleoyltaurine | 1.06 |  |
| 1888 |  | Inositol Metabolism | myo-inositol | 1.02 |  |
| 1889 |  |  | chiro-inositol | 0.64 |  |
| 1917 |  | Phospholipid Metabolism | choline | 0.89 |  |
| 1918 |  |  | choline phosphate | 1.21 |  |
| 1921 |  |  | glycerophosphorylcholine (GPC) | **1.26** |  |
| 1923 |  |  | phosphoethanolamine | 1.37 |  |
| 1925 |  |  | glycerophosphoethanolamine | **1.19** |  |
| 1927 |  |  | glycerophosphoinositol* | **3.89** |  |
| 1928 |  |  | trimethylamine N-oxide | 0.81 |  |
| 2170 |  | Phosphatidylserine (PS) | 1-stearoyl-2-oleoyl-GPS (18:0/18:1) | **0.66** |  |
| 2173 |  |  | 1-stearoyl-2-arachidonoyl-GPS (18:0/20:4) | 0.81 |  |
| 2190 |  | Phosphatidylglycerol (PG) | 1-stearoyl-2-oleoyl-GPG (18:0/18:1) | 0.89 |  |
| 2270 |  | Lysophospholipid | 1-linoleoyl-GPA (18:2)* | 1.06 |  |
| 2371 |  |  | 1-oleoyl-GPS (18:1) | 0.57 |  |
| 2376 |  |  | 1-palmitoyl-GPG (16:0)* | 0.95 |  |
| 2380 |  |  | 1-oleoyl-GPG (18:1)* | 0.78 |  |
| 2382 |  |  | 1-linoleoyl-GPG (18:2)* | 0.87 |  |
| 2503 |  | Glycerolipid Metabolism | glycerol | 1.23 |  |
| 2504 |  |  | glycerol 3-phosphate | **0.71** |  |
| 2509 |  |  | glycerophosphoglycerol | 1.37 |  |
| 2627 |  | Sphingolipid Synthesis | sphinganine | **0.72** |  |
| 2628 |  |  | sphinganine-1-phosphate | 1.07 |  |
| 2629 |  |  | sphingadienine | 0.78 |  |
| 2753 |  | Ceramide PEs | palmitoyl-sphingosine-phosphoethanolamine (d18:1/16:0) | 1.13 |  |
| 2778 |  | Sphingosines | sphingosine | **0.64** |  |
| 2780 |  |  | sphingosine 1-phosphate | 0.95 |  |
| 2796 |  | Mevalonate Metabolism | 3-hydroxy-3-methylglutarate | **1.76** |  |
| 2811 |  | Sterol | cholesterol | 0.94 |  |
| 2822 |  |  | 7-alpha-hydroxy-3-oxo-4-cholestenoate (7-Hoca) | 1.16 |  |
| 2824 |  |  | 3beta,7alpha-dihydroxy-5-cholestenoate | 0.97 |  |
| 2825 |  |  | 3beta-hydroxy-5-cholestenoate | 0.96 |  |
| 2835 |  |  | 4-cholesten-3-one | 1.01 |  |
| 2836 |  |  | beta-sitosterol | 1.08 |  |
| 2839 |  |  | campesterol | 1.20 |  |
| 2858 |  | Pregnenolone Steroids | pregnenolone sulfate | 0.86 |  |
| 2863 |  |  | 17alpha-hydroxypregnanolone glucuronide | 1.32 |  |
| 2868 |  |  | 21-hydroxypregnenolone disulfate | 1.02 |  |
| 2874 |  |  | pregnenediol sulfate (C21H34O5S)* | 0.90 |  |
| 2875 |  |  | pregnenediol disulfate (C21H34O8S2)* | 0.87 |  |
| 2876 |  |  | pregnenetriol sulfate* | **0.65** |  |
| 2877 |  |  | pregnenetriol disulfate* | 0.71 |  |
| 2886 |  | Progestin Steroids | 5alpha-pregnan-3beta,20beta-diol monosulfate (1) | 0.69 |  |
| 2889 |  |  | 5alpha-pregnan-3beta,20alpha-diol monosulfate (2) | 0.81 |  |
| 2895 |  |  | 5alpha-pregnan-3beta,20alpha-diol disulfate | 0.72 |  |
| 2898 |  |  | pregnanediol-3-glucuronide | 1.14 |  |
| 2918 |  | Corticosteroids | cortisol | 0.78 |  |
| 2920 |  |  | cortisone | 0.77 |  |
| 2926 |  |  | cortolone glucuronide (1) | **1.33** |  |
| 2939 |  | Androgenic Steroids | dehydroepiandrosterone sulfate (DHEA-S) | **0.56** |  |
| 2940 |  |  | 16a-hydroxy DHEA 3-sulfate | 0.65 |  |
| 2941 |  |  | androsterone glucuronide | 1.30 |  |
| 2943 |  |  | epiandrosterone sulfate | 0.66 |  |
| 2951 |  |  | androsterone sulfate | 0.68 |  |
| 2954 |  |  | etiocholanolone glucuronide | 1.12 |  |
| 2957 |  |  | androstenediol (3beta,17beta) monosulfate (1) | **0.46** |  |
| 2958 |  |  | androstenediol (3beta,17beta) monosulfate (2) | 0.78 |  |
| 2960 |  |  | androstenediol (3beta,17beta) disulfate (1) | **0.58** |  |
| 2961 |  |  | androstenediol (3beta,17beta) disulfate (2) | 0.68 |  |
| 2965 |  |  | androstenediol (3alpha, 17alpha) monosulfate (3) | 0.69 |  |
| 2974 |  |  | 5alpha-androstan-3alpha,17beta-diol monosulfate (1) | 0.49 |  |
| 2975 |  |  | 5alpha-androstan-3alpha,17beta-diol monosulfate (2) | 1.13 |  |
| 2976 |  |  | 5alpha-androstan-3alpha,17beta-diol disulfate | 0.87 |  |
| 2982 |  |  | 5alpha-androstan-3beta,17beta-diol disulfate | 0.66 |  |
| 2985 |  |  | 5alpha-androstan-3beta,17alpha-diol disulfate | 0.71 |  |
| 2997 |  |  | 11beta-hydroxyandrosterone glucuronide | 0.83 |  |
| 3016 |  | Primary Bile Acid Metabolism | cholate | 1.01 |  |
| 3017 |  |  | glycocholate | 0.55 |  |
| 3018 |  |  | taurocholate | 0.32 |  |
| 3019 |  |  | chenodeoxycholate | 0.89 |  |
| 3020 |  |  | glycochenodeoxycholate | 0.54 |  |
| 3021 |  |  | taurochenodeoxycholate | 0.59 |  |
| 3028 |  |  | glyco-beta-muricholate** | 1.74 |  |
| 3031 |  |  | glycochenodeoxycholate glucuronide (1) | 0.90 |  |
| 3033 |  |  | glycochenodeoxycholate 3-sulfate | 0.40 |  |
| 3037 |  | Secondary Bile Acid Metabolism | deoxycholate | 1.22 |  |
| 3041 |  |  | glycodeoxycholate | 1.21 |  |
| 3042 |  |  | taurodeoxycholate | 0.72 |  |
| 3043 |  |  | taurodeoxycholic acid 3-sulfate | 0.62 |  |
| 3049 |  |  | lithocholate sulfate (1) | 1.43 |  |
| 3050 |  |  | lithocholic acid sulfate (2) | 1.70 |  |
| 3051 |  |  | glycolithocholate | 1.18 |  |
| 3052 |  |  | glycolithocholate sulfate* | 0.83 |  |
| 3054 |  |  | taurolithocholate 3-sulfate | 0.49 |  |
| 3056 |  |  | ursodeoxycholate | 0.57 |  |
| 3058 |  |  | isoursodeoxycholate | 0.39 |  |
| 3059 |  |  | glycoursodeoxycholate | 0.32 |  |
| 3061 |  |  | tauroursodeoxycholate | 0.32 |  |
| 3068 |  |  | hyocholate | 1.11 |  |
| 3078 |  |  | glycocholenate sulfate* | 0.84 |  |
| 3079 |  |  | taurocholenate sulfate* | **0.60** |  |
| 3083 |  |  | glycodeoxycholate 3-sulfate | 0.79 |  |
| 3084 |  |  | taurochenodeoxycholic acid 3-sulfate | 0.30 |  |
| 3088 |  |  | glycodeoxycholate glucuronide (1) | 1.35 |  |
| 3656 | Nucleotide | Purine Metabolism, (Hypo)Xanthine/Inosine containing | inosine | 0.79 |  |
| 3657 |  |  | hypoxanthine | 0.89 |  |
| 3658 |  |  | xanthine | **0.54** |  |
| 3660 |  |  | xanthosine | 0.88 |  |
| 3663 |  |  | N1-methylinosine | **2.06** |  |
| 3666 |  |  | urate | **0.86** |  |
| 3668 |  |  | allantoin | 0.98 |  |
| 3674 |  | Purine Metabolism, Adenine containing | adenosine 5'-monophosphate (AMP) | 0.94 |  |
| 3682 |  |  | adenine | 1.31 |  |
| 3687 |  |  | N1-methyladenosine | **1.10** |  |
| 3697 |  |  | N6-carbamoylthreonyladenosine | **1.87** |  |
| 3707 |  |  | N6-succinyladenosine | **1.43** |  |
| 3718 |  | Purine Metabolism, Guanine containing | 7-methylguanine | 1.01 |  |
| 3724 |  |  | N2,N2-dimethylguanosine | **1.62** |  |
| 3736 |  | Pyrimidine Metabolism, Orotate containing | dihydroorotate | 0.91 |  |
| 3737 |  |  | orotate | **0.45** |  |
| 3739 |  |  | orotidine | 0.14 |  |
| 3744 |  | Pyrimidine Metabolism, Uracil containing | uridine 5'-monophosphate (UMP) | **1.98** |  |
| 3746 |  |  | uridine 3'-monophosphate (3'-UMP) | 1.57 |  |
| 3749 |  |  | uridine | **1.15** |  |
| 3750 |  |  | uracil | 0.96 |  |
| 3751 |  |  | pseudouridine | **1.25** |  |
| 3752 |  |  | 5,6-dihydrouridine | **1.28** |  |
| 3754 |  |  | 5-methyluridine (ribothymidine) | 1.00 |  |
| 3761 |  |  | 5,6-dihydrouracil | 0.93 |  |
| 3765 |  |  | 2'-deoxyuridine | **0.78** |  |
| 3768 |  |  | 3-ureidopropionate | **1.46** |  |
| 3769 |  |  | beta-alanine | 1.01 |  |
| 3770 |  |  | N-acetyl-beta-alanine | **1.12** |  |
| 3773 |  |  | 3-(3-amino-3-carboxypropyl)uridine* | **1.62** |  |
| 3776 |  | Pyrimidine Metabolism, Cytidine containing | cytidine 5'-monophosphate (5'-CMP) | 1.12 |  |
| 3780 |  |  | cytidine | **2.84** |  |
| 3781 |  |  | cytosine | 1.11 |  |
| 3782 |  |  | 3-methylcytidine | **1.20** |  |
| 3784 |  |  | N4-acetylcytidine | **1.54** |  |
| 3791 |  |  | 2'-O-methylcytidine | **1.35** |  |
| 3802 |  | Pyrimidine Metabolism, Thymine containing | thymine | **0.77** |  |
| 3803 |  |  | 5,6-dihydrothymine | **0.65** |  |
| 3805 |  |  | 3-aminoisobutyrate | **1.25** |  |
| 3823 | Cofactors and Vitamins | Nicotinate and Nicotinamide Metabolism | quinolinate | 1.19 |  |
| 3827 |  |  | nicotinamide | 1.19 |  |
| 3830 |  |  | nicotinamide riboside | 1.14 |  |
| 3839 |  |  | 1-methylnicotinamide | 0.74 |  |
| 3844 |  |  | trigonelline (N'-methylnicotinate) | 1.29 |  |
| 3846 |  |  | N1-Methyl-2-pyridone-5-carboxamide | 0.74 |  |
| 3848 |  |  | adenosine 5'-diphosphoribose (ADP-ribose) | 1.08 |  |
| 3851 |  | Riboflavin Metabolism | riboflavin (Vitamin B2) | 1.00 |  |
| 3854 |  | Pantothenate and CoA Metabolism | pantoate | 0.74 |  |
| 3855 |  |  | pantothenate | 1.09 |  |
| 3868 |  | Ascorbate and Aldarate Metabolism | threonate | 0.98 |  |
| 3871 |  |  | oxalate (ethanedioate) | 0.94 |  |
| 3872 |  |  | gulonate* | **1.66** |  |
| 3874 |  | Tocopherol Metabolism | alpha-tocopherol | 1.01 |  |
| 3878 |  |  | delta-tocopherol | 1.28 |  |
| 3882 |  |  | gamma-CEHC | 1.01 |  |
| 3883 |  |  | gamma-CEHC glucuronide* | **3.03** |  |
| 3885 |  |  | alpha-CEHC sulfate | 0.47 |  |
| 3896 |  |  | gamma-tocopherol/beta-tocopherol | 0.96 |  |
| 3918 |  | Hemoglobin and Porphyrin Metabolism | heme | 0.88 |  |
| 3919 |  |  | bilirubin (Z,Z) | 0.54 |  |
| 3920 |  |  | bilirubin (E,E)* | 0.82 |  |
| 3921 |  |  | bilirubin (E,Z or Z,E)* | 0.61 |  |
| 3922 |  |  | biliverdin | 0.79 |  |
| 3925 |  |  | L-urobilin | 0.46 |  |
| 3939 |  | Vitamin A Metabolism | retinol (Vitamin A) | 0.85 |  |
| 3941 |  |  | carotene diol (1) | 0.49 |  |
| 3942 |  |  | carotene diol (2) | 0.53 |  |
| 3943 |  |  | carotene diol (3) | **0.54** |  |
| 3945 |  |  | beta-cryptoxanthin | 0.96 |  |
| 3946 |  |  | retinal | **0.84** |  |
| 3959 |  | Vitamin B6 Metabolism | pyridoxate | **4.01** |  |
| 3961 | Xenobiotics | Benzoate Metabolism | hippurate | 2.42 |  |
| 3965 |  |  | 2-hydroxyhippurate (salicylurate) | 3.31 |  |
| 3967 |  |  | 3-hydroxyhippurate | 1.81 |  |
| 3968 |  |  | 3-hydroxyhippurate sulfate | 6.73 |  |
| 3969 |  |  | 4-hydroxyhippurate | 3.30 |  |
| 3975 |  |  | benzoate | 0.93 |  |
| 3985 |  |  | 4-hydroxybenzoate | **0.71** |  |
| 3993 |  |  | catechol sulfate | 0.94 |  |
| 3995 |  |  | 4-methylguaiacol sulfate | 1.08 |  |
| 3996 |  |  | guaiacol sulfate | 0.87 |  |
| 3997 |  |  | 3-methyl catechol sulfate (1) | 0.80 |  |
| 3999 |  |  | 4-ethylcatechol sulfate | 0.86 |  |
| 4000 |  |  | 4-methylcatechol sulfate | 0.74 |  |
| 4005 |  |  | methyl-4-hydroxybenzoate | **0.78** |  |
| 4013 |  |  | 4-ethylphenylsulfate | 1.15 |  |
| 4015 |  |  | 4-vinylphenol sulfate | 1.07 |  |
| 4027 |  |  | 3-methoxycatechol sulfate (1) | 1.34 |  |
| 4028 |  |  | 3-methoxycatechol sulfate (2) | 1.52 |  |
| 4030 |  |  | methyl-4-hydroxybenzoate sulfate | 0.99 |  |
| 4031 |  |  | propyl 4-hydroxybenzoate | **0.76** |  |
| 4037 |  |  | p-cresol sulfate | 0.66 |  |
| 4044 |  |  | o-cresol sulfate | 0.94 |  |
| 4048 |  |  | 3-(3-hydroxyphenyl)propionate sulfate | 1.37 |  |
| 4051 |  |  | 3-(3-hydroxyphenyl)propionate | 0.83 |  |
| 4055 |  | Xanthine Metabolism | caffeine | 1.21 |  |
| 4056 |  |  | paraxanthine | 0.97 |  |
| 4057 |  |  | theobromine | 0.77 |  |
| 4058 |  |  | theophylline | 0.88 |  |
| 4059 |  |  | 1-methylurate | 1.91 |  |
| 4060 |  |  | 7-methylurate | 2.41 |  |
| 4061 |  |  | 1,3-dimethylurate | **0.84** |  |
| 4062 |  |  | 1,7-dimethylurate | 1.29 |  |
| 4064 |  |  | 1,3,7-trimethylurate | 1.44 |  |
| 4065 |  |  | 1-methylxanthine | 0.89 |  |
| 4066 |  |  | 3-methylxanthine | 1.39 |  |
| 4067 |  |  | 7-methylxanthine | 0.88 |  |
| 4068 |  |  | 5-acetylamino-6-amino-3-methyluracil | 1.02 |  |
| 4069 |  |  | 5-acetylamino-6-formylamino-3-methyluracil | **0.53** |  |
| 4071 |  | Tobacco Metabolite | cotinine | 0.02 |  |
| 4072 |  |  | hydroxycotinine | 0.77 |  |
| 4073 |  |  | cotinine N-oxide | 0.81 |  |
| 4075 |  |  | 3-hydroxycotinine glucuronide | 0.79 |  |
| 4080 |  |  | norcotinine | 1.00 |  |
| 4081 |  |  | nornicotine | 0.98 |  |
| 4089 |  | Food Component/Plant | 2-piperidinone | 0.91 |  |
| 4162 |  |  | levulinate (4-oxovalerate) | 0.77 |  |
| 4164 |  |  | levulinoylcarnitine | **0.45** |  |
| 4173 |  |  | 1,6-anhydroglucose | 1.12 |  |
| 4175 |  |  | 2,3-dihydroxyisovalerate | 1.11 |  |
| 4183 |  |  | 2-isopropylmalate | **3.50** |  |
| 4187 |  |  | 3-formylindole | **0.65** |  |
| 4204 |  |  | betonicine | 3.00 |  |
| 4208 |  |  | 5-hydroxymethyl-2-furoylcarnitine* | 0.63 |  |
| 4209 |  |  | gluconate | 0.99 |  |
| 4220 |  |  | alliin | 0.57 |  |
| 4221 |  |  | N-acetylalliin | 0.36 |  |
| 4232 |  |  | beta-guanidinopropanoate | 0.72 |  |
| 4238 |  |  | dihydrocaffeate sulfate (2) | 1.41 |  |
| 4256 |  |  | cinnamoylglycine | **2.69** |  |
| 4280 |  |  | dihydroferulate | 1.23 |  |
| 4290 |  |  | ergothioneine | **0.72** |  |
| 4292 |  |  | erythritol | 1.00 |  |
| 4294 |  |  | 3-ethylcatechol sulfate (1) | **0.61** |  |
| 4297 |  |  | ferulic acid 4-sulfate | **2.11** |  |
| 4321 |  |  | homostachydrine* | 0.85 |  |
| 4324 |  |  | indolin-2-one | 0.83 |  |
| 4343 |  |  | mannonate* | 1.01 |  |
| 4347 |  |  | methyl indole-3-acetate | **3.30** |  |
| 4350 |  |  | N-(2-furoyl)glycine | 3.38 |  |
| 4373 |  |  | phytanate | 0.88 |  |
| 4379 |  |  | piperine | **0.28** |  |
| 4382 |  |  | glucuronide of piperine metabolite C17H21NO3 (3)* | **0.47** |  |
| 4383 |  |  | glucuronide of piperine metabolite C17H21NO3 (4)* | 0.55 |  |
| 4384 |  |  | glucuronide of piperine metabolite C17H21NO3 (5)* | **0.47** |  |
| 4391 |  |  | sulfate of piperine metabolite C16H19NO3 (2)* | **0.33** |  |
| 4392 |  |  | sulfate of piperine metabolite C16H19NO3 (3)* | **0.29** |  |
| 4397 |  |  | sulfate of piperine metabolite C18H21NO3 (1)* | **0.31** |  |
| 4399 |  |  | sulfate of piperine metabolite C18H21NO3 (3)* | **0.34** |  |
| 4408 |  |  | quinate | 1.30 |  |
| 4413 |  |  | saccharin | **4.92** |  |
| 4415 |  |  | acesulfame | 1.72 |  |
| 4416 |  |  | S-allylcysteine | **0.42** |  |
| 4425 |  |  | solanidine | **1.32** |  |
| 4433 |  |  | stachydrine | 1.02 |  |
| 4434 |  |  | 3-hydroxystachydrine* | 0.80 |  |
| 4441 |  |  | tartarate | 2.24 |  |
| 4442 |  |  | theanine | 5.91 |  |
| 4445 |  |  | thymol sulfate | 1.35 |  |
| 4457 |  |  | 4-allylphenol sulfate | 0.63 |  |
| 4458 |  |  | ethyl alpha-glucopyranoside | 0.72 |  |
| 4459 |  |  | methyl glucopyranoside (alpha + beta) | 1.01 |  |
| 4486 |  |  | 4-vinylguaiacol sulfate | 1.01 |  |
| 4487 |  |  | pyrraline | 0.89 |  |
| 4492 |  |  | eugenol sulfate | 1.25 |  |
| 4496 |  |  | 2-keto-3-deoxy-gluconate | 0.89 |  |
| 4518 |  |  | 3,4-methyleneheptanoate | 1.17 |  |
| 4568 |  |  | caffeic acid sulfate | 1.57 |  |
| 4570 |  |  | tartronate (hydroxymalonate) | 0.93 |  |
| 4581 |  | Bacterial/Fungal | lactobacillic acid | 1.09 |  |
| 4610 |  |  | 1H-indole-7-acetic acid | 0.65 |  |
| 4611 |  |  | N-methylpipecolate | 1.11 |  |
| 4635 |  | Drug - Analgesics, Anesthetics | 4-acetamidophenol | 0.94 |  |
| 4636 |  |  | 3-(N-acetyl-L-cystein-S-yl) acetaminophen | 5.96 |  |
| 4637 |  |  | 4-acetaminophen sulfate | 2.12 |  |
| 4638 |  |  | 4-acetamidophenylglucuronide | 2.99 |  |
| 4639 |  |  | 2-hydroxyacetaminophen sulfate* | 1.47 |  |
| 4640 |  |  | 2-methoxyacetaminophen sulfate* | 3.67 |  |
| 4641 |  |  | 2-methoxyacetaminophen glucuronide* | 5.75 |  |
| 4642 |  |  | 3-(cystein-S-yl)acetaminophen* | 4.10 |  |
| 4646 |  |  | 3-(methylthio)acetaminophen sulfate* | 3.26 |  |
| 4647 |  |  | 2-acetamidophenol sulfate | **1.86** |  |
| 4649 |  |  | 4-aminophenol sulfate (2) | 6.43 |  |
| 4650 |  |  | naproxen | 2.04 |  |
| 4651 |  |  | desmethylnaproxen | 1.18 |  |
| 4652 |  |  | desmethylnaproxen sulfate | 3.24 |  |
| 4653 |  |  | ibuprofen | 7.90 |  |
| 4654 |  |  | 2-hydroxyibuprofen | 1.11 |  |
| 4656 |  |  | carboxyibuprofen | 0.76 |  |
| 4657 |  |  | carboxyibuprofen glucuronide* | 1.36 |  |
| 4658 |  |  | ibuprofen acyl glucuronide | 2.38 |  |
| 4662 |  |  | salicyluric glucuronide* | **8.33** |  |
| 4676 |  |  | lidocaine | 1.13 |  |
| 4677 |  |  | N-ethylglycinexylidide | 1.39 |  |
| 4686 |  |  | tramadol | 14.83 |  |
| 4687 |  |  | O-desmethyltramadol | 2.02 |  |
| 4688 |  |  | O-desmethyltramadol glucuronide | **1.10** |  |
| 4689 |  |  | N-desmethyl tramadol | **1.02** |  |
| 4712 |  | Drug - Antibiotic | azithromycin | 38.76 |  |
| 4725 |  |  | sulfamethoxazole | 1.00 |  |
| 4734 |  |  | linezolid | 1.00 |  |
| 4752 |  |  | N4-acetylsulfamethoxazole* | **23.77** |  |
| 4762 |  | Drug - Antiviral | nelfinavir | **1.30** |  |
| 4820 |  | Drug - Cardiovascular | metoprolol | 1.17 |  |
| 4821 |  |  | metoprolol acid metabolite* | 1.02 |  |
| 4822 |  |  | alpha-hydroxymetoprolol | 0.65 |  |
| 4825 |  |  | atenolol | 1.39 |  |
| 4832 |  |  | 4-hydroxycoumarin | 0.62 |  |
| 4836 |  |  | warfarin | 0.89 |  |
| 4837 |  |  | 7-hydroxywarfarin | 0.88 |  |
| 4838 |  |  | 10-hydroxywarfarin | 1.18 |  |
| 4840 |  |  | hydrochlorothiazide | **0.33** |  |
| 4841 |  |  | triamterene | 0.98 |  |
| 4842 |  |  | furosemide | 0.99 |  |
| 4843 |  |  | chlorthalidone | 0.98 |  |
| 4846 |  |  | valsartan | 1.00 |  |
| 4847 |  |  | olmesartan | 1.00 |  |
| 4859 |  | Drug - Gastrointestinal | ondansetron | 0.91 |  |
| 4862 |  |  | ranitidine | 1.07 |  |
| 4863 |  |  | ranitidine N-oxide* | 1.03 |  |
| 4864 |  |  | famotidine | 1.14 |  |
| 4866 |  |  | omeprazole | 0.56 |  |
| 4867 |  |  | pantoprazole | 0.36 |  |
| 4874 |  | Drug - Metabolic | metformin | **0.30** |  |
| 4897 |  |  | oxypurinol | 0.15 |  |
| 4904 |  | Drug - Neurological | valproate | 1.00 |  |
| 4905 |  |  | valproic acid glucuronide | 0.22 |  |
| 4907 |  |  | 3-hydroxyvalproate | 1.00 |  |
| 4908 |  |  | 2-propyl-2-pentenoate (2-ene-valproate) | 0.04 |  |
| 4909 |  |  | 2-propyl-4-pentenoate (4-ene-valproate) | 1.00 |  |
| 4919 |  |  | gabapentin | 1.03 |  |
| 4920 |  |  | lamotrigine | **347.83** |  |
| 4936 |  |  | pregabalin | 1.15 |  |
| 4943 |  | Drug - Psychoactive | hydroxybupropion | **253.43** |  |
| 4945 |  |  | venlafaxine | 1.00 |  |
| 4946 |  |  | O-desmethylvenlafaxine | 1.00 |  |
| 4955 |  |  | maprotiline | 0.99 |  |
| 4958 |  |  | escitalopram | 1.07 |  |
| 4968 |  |  | trazadone | 18.78 |  |
| 4974 |  |  | zolpidem | 0.97 |  |
| 4983 |  | Drug - Respiratory | diphenhydramine | 1.31 |  |
| 4987 |  |  | fexofenadine | 1.68 |  |
| 4988 |  |  | cetirizine | 0.04 |  |
| 4992 |  |  | guaifenesin | 1.08 |  |
| 4995 |  |  | pseudoephedrine | 1.24 |  |
| 4999 |  | Drug - Topical Agents | salicylate | 3.32 |  |
| 5000 |  |  | 2,6-dihydroxybenzoic acid | 0.76 |  |
| 5004 |  |  | hydroquinone sulfate | **4.59** |  |
| 5029 |  | Drug - Other | 1-hydroxy-2-naphthalenecarboxylate | 0.99 |  |
| 5077 |  | Chemical | sulfate* | 0.85 |  |
| 5080 |  |  | O-sulfo-L-tyrosine | 0.99 |  |
| 5099 |  |  | 2-aminophenol sulfate | 1.37 |  |
| 5141 |  |  | dexpanthenol | 1.00 |  |
| 5143 |  |  | dimethyl sulfone | 0.80 |  |
| 5145 |  |  | ectoine | 1.10 |  |
| 5146 |  |  | EDTA | **0.81** |  |
| 5151 |  |  | 2-acrylamidoglycolic acid | 1.18 |  |
| 5155 |  |  | iminodiacetate (IDA) | **0.74** |  |
| 5174 |  |  | benzoylcarnitine* | 0.77 |  |
| 5175 |  |  | perfluorooctanesulfonate (PFOS) | **0.56** |  |
| 5184 |  |  | succinimide | 0.89 |  |
| 5192 |  |  | 4-methylbenzenesulfonate | 0.20 |  |
| 5195 |  |  | 4-hydroxychlorothalonil | **0.64** |  |
| 5199 |  |  | 1,2,3-benzenetriol sulfate (2) | 1.19 |  |
| 5201 |  |  | 2-methoxyresorcinol sulfate | **1.71** |  |
| 5202 |  |  | 3-hydroxypyridine sulfate | 1.08 |  |
| 5203 |  |  | 3-hydroxypyridine glucuronide | **4.19** |  |
| 5208 |  |  | ethylparaben sulfate | 1.09 |  |
| 5213 |  |  | 6-hydroxyindole sulfate | 0.88 |  |
| 5217 |  |  | thioproline | **0.75** |  |
| 5219 |  |  | perfluorooctanoate (PFOA) | **0.61** |  |
| 5229 |  |  | 2-hydroxy-5-methylpyridine sulfate | 1.87 |  |
| 5233 |  |  | 6-acetylglucose | 1.08 |  |
| 5257 | Partially Characterized Molecules | Partially Characterized Molecules | glucuronide of C10H18O2 (1)* | **1.89** |  |
| 5263 |  |  | glucuronide of C10H18O2 (7)* | **3.83** |  |
| 5293 |  |  | glycine conjugate of C10H14O2 (1)* | 1.27 |  |
| 5297 |  |  | metabolonic lactone sulfate | 0.70 |  |
