# Supplementary material for: Metabolomic and Lipidomic Profiling of Bone Marrow Plasma Differentiates Patients with Monoclonal Gammopathy of Undetermined Significance from Multiple Myeloma
Source: Sci Rep. 2020 Jun 24;10:10250. doi: 10.1038/s41598-020-67105-3 (PMC7314797; doi:10.1038/s41598-020-67105-3)
Supplement: Supplementary file 3 — Supplemental information3. [file 41598_2020_67105_MOESM3_ESM.docx]

|  | **SUPPLEMENTARY TABLE 3: Red** and **green** shaded cells indicate p≤0.05 (**red** indicates that the mean values are significantly higher for that comparison; **green** values significantly lower). **Light red** and **light green** shaded cells indicate 0.05<p<0.10 (**light red** indicates that the mean values trend higher for that comparison; l**ight green** values trend lower). | | |  |  |
| --- | --- | --- | --- | --- | --- |
|  |  |  |  | **Fold of Change** |  |
|  |  |  |  | **MM** |  |
|  |  |  |  |  |  |
|  | **Pathway Sort Order** | **Super Pathway** | **Sub Pathway** | **MGUS** |  |
|  | 6543 | Phospholipid SUM | Phosphatidylcholines | 0.91 |  |
|  | 6544 |  | Lysophosphatidylcholines | 0.90 |  |
|  | 6545 |  | Phosphatidylethanolamines | **0.83** |  |
|  | 6546 |  | Lysophosphatidylethanolamines | 0.99 |  |
|  | 6547 |  | Phosphatidylinositols | 0.88 |  |
|  | 6548 | Sphingolipid SUM | Ceramides | 0.98 |  |
|  | 6549 |  | Dihydroceramides | 0.94 |  |
|  | 6550 |  | Hexosylceramides | 1.01 |  |
|  | 6551 |  | Lactosylceramides | **0.81** |  |
|  | 6552 |  | Sphingomyelins | 1.00 |  |
|  | 6554 | Neutral Complex Lipids SUM | Free Fatty Acids | 1.00 |  |
|  | 6555 |  | Cholesteryl Esters | 0.90 |  |
|  | 6556 |  | Diacylglycerols | 0.85 |  |
|  | 6558 |  | Triacylglycerols | 0.87 |  |
|  | 6559 |  | Monoacylglycerols | 0.93 |  |
|  | 6562 | Phosphatidylcholine | PC Ester | 0.95 |  |
|  | 6563 |  |  | 0.79 |  |
|  | 6575 |  |  | 0.73 |  |
|  | 6577 |  |  | **0.82** |  |
|  | 6578 |  |  | **0.76** |  |
|  | 6579 |  |  | **0.75** |  |
|  | 6580 |  |  | **0.70** |  |
|  | 6583 |  |  | 0.88 |  |
|  | 6584 |  |  | **0.69** |  |
|  | 6585 |  |  | **0.79** |  |
|  | 6586 |  |  | 0.79 |  |
|  | 6589 |  |  | 0.94 |  |
|  | 6590 |  |  | 0.88 |  |
|  | 6591 |  |  | 0.87 |  |
|  | 6593 |  |  | 0.85 |  |
|  | 6594 |  |  | **0.83** |  |
|  | 6595 |  |  | **0.81** |  |
|  | 6597 |  |  | **0.78** |  |
|  | 6598 |  |  | **0.83** |  |
|  | 6599 |  |  | 0.97 |  |
|  | 6602 |  |  | 0.94 |  |
|  | 6603 |  |  | **0.84** |  |
|  | 6604 |  |  | **0.77** |  |
|  | 6605 |  |  | 0.92 |  |
|  | 6606 |  |  | 1.03 |  |
|  | 6607 |  |  | **0.82** |  |
|  | 6608 |  |  | 0.99 |  |
|  | 6609 |  |  | 0.89 |  |
|  | 6610 |  |  | 0.90 |  |
|  | 6611 |  |  | **0.78** |  |
|  | 6612 |  |  | 0.99 |  |
|  | 6613 |  |  | 0.90 |  |
|  | 6614 |  |  | **0.82** |  |
|  | 6615 |  |  | **0.77** |  |
|  | 6616 |  |  | 0.96 |  |
|  | 6617 |  |  | **0.82** |  |
|  | 6620 |  |  | 0.80 |  |
|  | 6621 |  |  | 0.90 |  |
|  | 6622 |  |  | 1.05 |  |
|  | 6624 |  |  | **0.85** |  |
|  | 6625 |  |  | 0.90 |  |
|  | 6626 |  |  | 0.90 |  |
|  | 6627 |  |  | 0.94 |  |
|  | 6628 |  |  | **0.81** |  |
|  | 6629 |  |  | 1.00 |  |
|  | 6630 |  |  | 0.95 |  |
|  | 6632 |  |  | **0.92** |  |
|  | 6633 |  |  | 1.01 |  |
|  | 6634 |  |  | **0.85** |  |
|  | 6635 |  |  | **0.84** |  |
|  | 6637 |  |  | **0.75** |  |
|  | 6638 |  |  | 0.87 |  |
|  | 6639 |  |  | 0.85 |  |
|  | 6640 |  |  | 0.86 |  |
|  | 6641 |  |  | **0.72** |  |
|  | 6643 |  |  | 0.98 |  |
|  | 6644 |  |  | **0.83** |  |
|  | 6645 |  |  | **0.79** |  |
|  | 6646 |  |  | **0.75** |  |
|  | 6647 |  |  | 0.99 |  |
|  | 6648 |  |  | **0.79** |  |
|  | 6651 |  |  | 0.77 |  |
|  | 6652 |  |  | 0.83 |  |
|  | 6653 |  |  | 1.00 |  |
|  | 6655 |  |  | 0.92 |  |
|  | 6656 |  |  | 0.98 |  |
|  | 6657 |  |  | 0.93 |  |
|  | 6658 |  |  | 0.85 |  |
|  | 6660 |  |  | 0.97 |  |
|  | 6661 |  |  | 0.88 |  |
|  | 6662 |  |  | 0.81 |  |
|  | 6663 |  |  | 1.00 |  |
|  | 6664 |  |  | **0.84** |  |
|  | 6667 |  |  | 0.98 |  |
|  | 6668 |  |  | 0.96 |  |
|  | 6669 |  |  | 1.09 |  |
|  | 6671 |  |  | 0.87 |  |
|  | 6672 |  |  | 0.81 |  |
|  | 6673 |  |  | 0.95 |  |
|  | 6675 |  |  | 1.00 |  |
|  | 6676 |  |  | 0.88 |  |
|  | 6677 |  |  | **0.77** |  |
|  | 6678 |  |  | 0.83 |  |
|  | 6679 |  |  | 0.87 |  |
|  | 6682 |  |  | 0.97 |  |
|  | 6683 |  |  | 0.99 |  |
|  | 6684 |  |  | 1.04 |  |
|  | 6685 |  |  | 0.94 |  |
|  | 6686 |  |  | 0.89 |  |
|  | 6687 |  |  | 0.88 |  |
|  | 6688 |  |  | 0.88 |  |
|  | 6689 |  |  | 1.03 |  |
|  | 6693 |  |  | **0.77** |  |
|  | 6694 |  |  | 0.97 |  |
|  | 6695 |  |  | 0.98 |  |
|  | 6698 |  |  | 1.00 |  |
|  | 6699 |  |  | 1.01 |  |
|  | 6782 | Lysophosphatidylcholine | LPC Ester | **0.71** |  |
|  | 6784 |  |  | **0.79** |  |
|  | 6785 |  |  | 0.88 |  |
|  | 6786 |  |  | **0.85** |  |
|  | 6787 |  |  | 0.93 |  |
|  | 6788 |  |  | 0.92 |  |
|  | 6789 |  |  | 0.95 |  |
|  | 6790 |  |  | 0.90 |  |
|  | 6791 |  |  | **0.79** |  |
|  | 6793 |  |  | 0.99 |  |
|  | 6794 |  |  | 1.08 |  |
|  | 6795 |  |  | 0.97 |  |
|  | 6796 |  |  | 0.81 |  |
|  | 6797 |  |  | 1.03 |  |
|  | 6798 |  |  | 0.88 |  |
|  | 6802 |  |  | 0.95 |  |
|  | 6803 |  |  | 1.04 |  |
|  | 6804 |  |  | 1.12 |  |
|  | 6849 | Phosphatidylethanolamine | PE Ester | 0.94 |  |
|  | 6850 |  |  | **0.77** |  |
|  | 6853 |  |  | 0.89 |  |
|  | 6854 |  |  | 0.87 |  |
|  | 6855 |  |  | **0.78** |  |
|  | 6857 |  |  | 0.95 |  |
|  | 6858 |  |  | 0.96 |  |
|  | 6859 |  |  | 0.77 |  |
|  | 6860 |  |  | 0.91 |  |
|  | 6861 |  |  | **0.84** |  |
|  | 6864 |  |  | 0.74 |  |
|  | 6865 |  |  | 0.81 |  |
|  | 6866 |  |  | 0.98 |  |
|  | 6874 |  |  | **0.92** |  |
|  | 6875 |  |  | 0.84 |  |
|  | 6878 |  |  | 0.90 |  |
|  | 6880 |  |  | 0.93 |  |
|  | 6884 |  |  | 1.01 |  |
|  | 6885 |  |  | 1.00 |  |
|  | 6886 |  |  | 0.88 |  |
|  | 6890 |  |  | 1.00 |  |
|  | 6891 |  |  | 0.95 |  |
|  | 6893 |  |  | 0.86 |  |
|  | 6894 |  |  | 0.88 |  |
|  | 6895 |  |  | 0.87 |  |
|  | 6896 |  |  | **0.79** |  |
|  | 6898 |  |  | 0.92 |  |
|  | 6899 |  |  | **0.84** |  |
|  | 6900 |  |  | **0.78** |  |
|  | 6901 |  |  | 0.95 |  |
|  | 6902 |  |  | 0.86 |  |
|  | 6905 |  |  | 0.77 |  |
|  | 6906 |  |  | 0.82 |  |
|  | 6907 |  |  | 0.98 |  |
|  | 6908 |  |  | 0.99 |  |
|  | 6909 |  |  | **0.84** |  |
|  | 6910 |  |  | 0.93 |  |
|  | 6911 |  |  | 0.98 |  |
|  | 6912 |  |  | 0.98 |  |
|  | 6914 |  |  | 1.00 |  |
|  | 6915 |  |  | **1.14** |  |
|  | 6916 |  |  | 0.85 |  |
|  | 6917 |  |  | 1.02 |  |
|  | 6918 |  |  | 1.01 |  |
|  | 6919 |  |  | 0.94 |  |
|  | 6922 |  |  | 0.88 |  |
|  | 6923 |  |  | 0.96 |  |
|  | 6924 |  |  | 1.00 |  |
|  | 6925 |  |  | 0.93 |  |
|  | 6926 |  |  | **0.67** |  |
|  | 6927 |  |  | 0.94 |  |
|  | 6931 |  |  | 1.00 |  |
|  | 6933 |  |  | 0.89 |  |
|  | 6939 |  |  | 1.00 |  |
|  | 6942 |  | PE Ether | 1.00 |  |
|  | 6945 |  |  | **0.78** |  |
|  | 6946 |  |  | **0.76** |  |
|  | 6951 |  |  | 0.78 |  |
|  | 6952 |  |  | 0.71 |  |
|  | 6953 |  |  | 0.98 |  |
|  | 6956 |  |  | 0.82 |  |
|  | 6957 |  |  | **0.73** |  |
|  | 6958 |  |  | 0.86 |  |
|  | 6960 |  |  | **0.80** |  |
|  | 6962 |  |  | 1.00 |  |
|  | 6963 |  |  | **0.81** |  |
|  | 6964 |  |  | **0.78** |  |
|  | 6969 |  |  | **0.71** |  |
|  | 6970 |  |  | 0.76 |  |
|  | 6971 |  |  | **0.71** |  |
|  | 6974 |  |  | 0.90 |  |
|  | 6975 |  |  | 0.80 |  |
|  | 6976 |  |  | 0.86 |  |
|  | 6981 |  | PE Plasmalogen | **0.82** |  |
|  | 6982 |  |  | 0.94 |  |
|  | 6983 |  |  | **0.84** |  |
|  | 6984 |  |  | **0.78** |  |
|  | 6985 |  |  | **0.71** |  |
|  | 6986 |  |  | 0.92 |  |
|  | 6988 |  |  | 0.93 |  |
|  | 6989 |  |  | 0.93 |  |
|  | 6990 |  |  | **0.67** |  |
|  | 6991 |  |  | 0.73 |  |
|  | 6992 |  |  | **0.65** |  |
|  | 6995 |  |  | 0.86 |  |
|  | 6996 |  |  | **0.76** |  |
|  | 6997 |  |  | 0.87 |  |
|  | 6998 |  |  | **0.82** |  |
|  | 7000 |  |  | **0.75** |  |
|  | 7001 |  |  | **0.81** |  |
|  | 7002 |  |  | **0.80** |  |
|  | 7003 |  |  | **0.77** |  |
|  | 7004 |  |  | **0.72** |  |
|  | 7005 |  |  | **0.78** |  |
|  | 7007 |  |  | **0.90** |  |
|  | 7008 |  |  | **0.76** |  |
|  | 7009 |  |  | **0.67** |  |
|  | 7010 |  |  | 0.73 |  |
|  | 7011 |  |  | 0.67 |  |
|  | 7014 |  |  | 0.84 |  |
|  | 7015 |  |  | 0.78 |  |
|  | 7016 |  |  | 0.83 |  |
|  | 7018 |  |  | **0.78** |  |
|  | 7019 |  |  | 0.94 |  |
|  | 7020 |  |  | 0.99 |  |
|  | 7021 |  |  | 0.83 |  |
|  | 7022 |  |  | **0.74** |  |
|  | 7023 |  |  | **0.92** |  |
|  | 7026 |  |  | **0.94** |  |
|  | 7027 |  |  | **0.72** |  |
|  | 7028 |  |  | **0.75** |  |
|  | 7029 |  |  | 0.73 |  |
|  | 7032 |  |  | 0.95 |  |
|  | 7033 |  |  | 0.82 |  |
|  | 7034 |  |  | 0.89 |  |
|  | 7035 |  |  | 0.84 |  |
|  | 7036 |  |  | 0.80 |  |
|  | 7037 |  |  | 0.99 |  |
|  | 7041 | Lysophosphatidylethanolamine | LPE Ester | 1.04 |  |
|  | 7042 |  |  | 1.04 |  |
|  | 7043 |  |  | **0.77** |  |
|  | 7044 |  |  | 1.03 |  |
|  | 7045 |  |  | 1.05 |  |
|  | 7046 |  |  | 0.96 |  |
|  | 7047 |  |  | 0.94 |  |
|  | 7048 |  |  | 0.96 |  |
|  | 7051 |  |  | 1.00 |  |
|  | 7052 |  |  | **1.13** |  |
|  | 7053 |  |  | 0.86 |  |
|  | 7054 |  |  | 1.00 |  |
|  | 7055 |  |  | 0.86 |  |
|  | 7059 |  |  | 0.90 |  |
|  | 7060 |  |  | 0.93 |  |
|  | 7061 |  |  | 1.16 |  |
|  | 7315 | Phosphatidylinositol | PI Ester | **0.77** |  |
|  | 7316 |  |  | **0.71** |  |
|  | 7318 |  |  | **0.79** |  |
|  | 7319 |  |  | **0.83** |  |
|  | 7320 |  |  | 1.00 |  |
|  | 7324 |  |  | **0.71** |  |
|  | 7325 |  |  | **0.75** |  |
|  | 7329 |  |  | **0.83** |  |
|  | 7330 |  |  | **0.77** |  |
|  | 7331 |  |  | 0.98 |  |
|  | 7350 |  |  | **0.87** |  |
|  | 7351 |  |  | **0.80** |  |
|  | 7354 |  |  | **0.82** |  |
|  | 7355 |  |  | 0.97 |  |
|  | 7356 |  |  | 0.93 |  |
|  | 7359 |  |  | **0.82** |  |
|  | 7360 |  |  | 0.84 |  |
|  | 7361 |  |  | 0.90 |  |
|  | 7362 |  |  | 0.97 |  |
|  | 7364 |  |  | 0.89 |  |
|  | 7365 |  |  | 0.85 |  |
|  | 7366 |  |  | 0.98 |  |
|  | 7368 |  |  | 0.84 |  |
|  | 7369 |  |  | 0.79 |  |
|  | 7370 |  |  | 0.93 |  |
|  | 7375 |  |  | 0.83 |  |
|  | 7376 |  |  | 0.87 |  |
|  | 7386 |  |  | **1.11** |  |
|  | 7713 | Free Fatty Acids | Saturated FFA | 1.09 |  |
|  | 7715 |  |  | 1.01 |  |
|  | 7716 |  |  | 1.04 |  |
|  | 7717 |  |  | 0.97 |  |
|  | 7718 |  |  | 1.00 |  |
|  | 7719 |  |  | 0.99 |  |
|  | 7721 |  |  | 1.05 |  |
|  | 7723 |  |  | 1.08 |  |
|  | 7725 |  |  | 0.87 |  |
|  | 7726 |  | Monounsaturated FFA | 1.00 |  |
|  | 7727 |  |  | 1.03 |  |
|  | 7728 |  |  | 1.01 |  |
|  | 7729 |  |  | 0.81 |  |
|  | 7730 |  |  | **1.22** |  |
|  | 7731 |  |  | **1.18** |  |
|  | 7733 |  | Polyunsaturated FFA (PUFA) | 1.03 |  |
|  | 7735 |  |  | 1.03 |  |
|  | 7736 |  |  | 0.75 |  |
|  | 7737 |  |  | 0.97 |  |
|  | 7738 |  |  | 0.99 |  |
|  | 7739 |  |  | 1.00 |  |
|  | 7740 |  |  | 1.11 |  |
|  | 7741 |  |  | 1.01 |  |
|  | 7742 |  |  | 1.01 |  |
|  | 7743 |  |  | 1.01 |  |
|  | 7744 | Cholesterol Ester | CE Ester | 0.72 |  |
|  | 7745 |  |  | **0.75** |  |
|  | 7746 |  |  | **0.64** |  |
|  | 7747 |  |  | 0.85 |  |
|  | 7748 |  |  | 0.94 |  |
|  | 7749 |  |  | **0.79** |  |
|  | 7750 |  |  | 0.87 |  |
|  | 7751 |  |  | 0.86 |  |
|  | 7752 |  |  | 0.89 |  |
|  | 7753 |  |  | 0.92 |  |
|  | 7754 |  |  | **0.74** |  |
|  | 7755 |  |  | **0.64** |  |
|  | 7756 |  |  | 0.81 |  |
|  | 7757 |  |  | 0.88 |  |
|  | 7758 |  |  | 0.87 |  |
|  | 7759 |  |  | 0.87 |  |
|  | 7760 |  |  | 0.97 |  |
|  | 7761 |  |  | 0.83 |  |
|  | 7762 |  |  | 0.82 |  |
|  | 7763 |  |  | 1.05 |  |
|  | 7764 |  |  | 0.95 |  |
|  | 7765 |  |  | 0.94 |  |
|  | 7766 |  |  | 0.93 |  |
|  | 7767 |  |  | 1.00 |  |
|  | 7768 |  |  | 0.94 |  |
|  | 7769 |  |  | 0.97 |  |
|  | 7770 | Sphingolipids | Ceramide | 1.02 |  |
|  | 7771 |  |  | 1.15 |  |
|  | 7772 |  |  | 1.39 |  |
|  | 7774 |  |  | 1.23 |  |
|  | 7775 |  |  | 1.20 |  |
|  | 7776 |  |  | 1.12 |  |
|  | 7777 |  |  | 0.98 |  |
|  | 7778 |  |  | 1.06 |  |
|  | 7779 |  |  | 0.86 |  |
|  | 7780 |  |  | 1.10 |  |
|  | 7781 |  |  | 0.92 |  |
|  | 7782 |  |  | 1.02 |  |
|  | 7788 |  | Dihydroceramide | 0.93 |  |
|  | 7789 |  |  | 1.07 |  |
|  | 7790 |  |  | 1.20 |  |
|  | 7791 |  |  | 1.00 |  |
|  | 7792 |  |  | 1.04 |  |
|  | 7793 |  |  | 0.84 |  |
|  | 7794 |  |  | 0.95 |  |
|  | 7795 |  |  | 0.98 |  |
|  | 7796 |  |  | 0.83 |  |
|  | 7797 |  |  | 0.86 |  |
|  | 7798 |  |  | 0.98 |  |
|  | 7799 |  |  | 0.86 |  |
|  | 7800 |  |  | 1.07 |  |
|  | 7801 |  | Hexosylceramide | 1.08 |  |
|  | 7802 |  |  | 1.10 |  |
|  | 7803 |  |  | 1.10 |  |
|  | 7804 |  |  | 1.12 |  |
|  | 7805 |  |  | 1.02 |  |
|  | 7806 |  |  | 1.09 |  |
|  | 7807 |  |  | 0.90 |  |
|  | 7808 |  |  | 1.09 |  |
|  | 7809 |  |  | 0.92 |  |
|  | 7810 |  |  | 1.07 |  |
|  | 7811 |  |  | 0.94 |  |
|  | 7812 |  |  | 1.02 |  |
|  | 7813 |  | Lactosylceramide | **0.83** |  |
|  | 7814 |  |  | **0.75** |  |
|  | 7815 |  |  | 1.07 |  |
|  | 7816 |  |  | 0.98 |  |
|  | 7817 |  |  | **1.17** |  |
|  | 7818 |  |  | 1.08 |  |
|  | 7819 |  |  | 1.02 |  |
|  | 7820 |  |  | **1.16** |  |
|  | 7821 |  |  | 0.89 |  |
|  | 7822 |  |  | 0.82 |  |
|  | 7823 |  |  | 1.01 |  |
|  | 7824 |  |  | 0.85 |  |
|  | 7825 |  | Sphingomyelin | 0.93 |  |
|  | 7826 |  |  | 1.03 |  |
|  | 7827 |  |  | 1.12 |  |
|  | 7828 |  |  | 1.07 |  |
|  | 7829 |  |  | 0.94 |  |
|  | 7830 |  |  | 0.98 |  |
|  | 7831 |  |  | 0.91 |  |
|  | 7832 |  |  | 0.92 |  |
|  | 7833 |  |  | 0.95 |  |
|  | 7834 |  |  | 1.04 |  |
|  | 7835 |  |  | 0.98 |  |
|  | 7836 |  |  | 1.08 |  |
|  | 7837 | Diacylglycerol | DAG Ester | 1.16 |  |
|  | 7839 |  |  | 0.96 |  |
|  | 7840 |  |  | 1.04 |  |
|  | 7841 |  |  | 0.96 |  |
|  | 7844 |  |  | 0.79 |  |
|  | 7846 |  |  | **0.66** |  |
|  | 7847 |  |  | **0.72** |  |
|  | 7850 |  |  | **0.71** |  |
|  | 7851 |  |  | 0.71 |  |
|  | 7852 |  |  | 0.73 |  |
|  | 7854 |  |  | **0.82** |  |
|  | 7855 |  |  | 0.67 |  |
|  | 7856 |  |  | **0.80** |  |
|  | 7857 |  |  | **0.86** |  |
|  | 7858 |  |  | 1.00 |  |
|  | 7864 |  |  | 0.74 |  |
|  | 7865 |  |  | 0.78 |  |
|  | 7870 |  |  | 0.80 |  |
|  | 7871 |  |  | **0.70** |  |
|  | 7873 |  |  | 0.77 |  |
|  | 7874 |  |  | 0.79 |  |
|  | 7875 |  |  | 0.90 |  |
|  | 7876 |  |  | 0.77 |  |
|  | 7879 |  |  | **0.73** |  |
|  | 7880 |  |  | 0.82 |  |
|  | 7881 |  |  | **0.75** |  |
|  | 7882 |  |  | 0.90 |  |
|  | 7883 |  |  | 0.85 |  |
|  | 7884 |  |  | 0.84 |  |
|  | 7885 |  |  | 0.89 |  |
|  | 7886 |  |  | 0.90 |  |
|  | 7887 |  |  | 0.79 |  |
|  | 7888 |  |  | 0.78 |  |
|  | 7889 |  |  | 0.89 |  |
|  | 7890 |  |  | 0.83 |  |
|  | 7891 |  |  | 0.70 |  |
|  | 7892 |  |  | 0.91 |  |
|  | 7898 |  |  | 0.76 |  |
|  | 7899 |  |  | 0.87 |  |
|  | 7900 |  |  | 0.86 |  |
|  | 7902 |  |  | 0.88 |  |
|  | 7903 |  |  | 0.82 |  |
|  | 7904 |  |  | 0.91 |  |
|  | 7905 |  |  | **0.79** |  |
|  | 7906 |  |  | **0.74** |  |
|  | 7907 |  |  | 0.70 |  |
|  | 7908 |  |  | 0.81 |  |
|  | 7909 |  |  | **0.75** |  |
|  | 7910 |  |  | **0.78** |  |
|  | 7911 |  |  | 0.84 |  |
|  | 7912 |  |  | 0.84 |  |
|  | 7914 |  |  | 0.88 |  |
|  | 7915 |  |  | 0.79 |  |
|  | 7916 |  |  | 0.86 |  |
|  | 7917 |  |  | 0.80 |  |
|  | 7918 |  |  | **0.73** |  |
|  | 7919 |  |  | 0.90 |  |
|  | 7920 |  |  | 0.84 |  |
|  | 8218 | Triacylglycerol | TAG Ester | 2.88 |  |
|  | 8219 |  |  | 1.71 |  |
|  | 8227 |  |  | 2.45 |  |
|  | 8229 |  |  | 0.94 |  |
|  | 8230 |  |  | 0.88 |  |
|  | 8248 |  |  | 1.21 |  |
|  | 8250 |  |  | 0.96 |  |
|  | 8252 |  |  | 0.93 |  |
|  | 8254 |  |  | 1.82 |  |
|  | 8256 |  |  | 0.75 |  |
|  | 8258 |  |  | **0.37** |  |
|  | 8259 |  |  | 0.81 |  |
|  | 8260 |  |  | 0.73 |  |
|  | 8261 |  |  | 1.90 |  |
|  | 8265 |  |  | 0.70 |  |
|  | 8288 |  |  | 1.29 |  |
|  | 8290 |  |  | **0.57** |  |
|  | 8292 |  |  | 1.02 |  |
|  | 8294 |  |  | 1.04 |  |
|  | 8296 |  |  | 0.99 |  |
|  | 8298 |  |  | **0.78** |  |
|  | 8299 |  |  | **0.57** |  |
|  | 8301 |  |  | 0.69 |  |
|  | 8302 |  |  | **0.81** |  |
|  | 8304 |  |  | 0.87 |  |
|  | 8306 |  |  | 0.91 |  |
|  | 8308 |  |  | 0.84 |  |
|  | 8310 |  |  | 0.59 |  |
|  | 8311 |  |  | **0.73** |  |
|  | 8312 |  |  | 0.58 |  |
|  | 8313 |  |  | 0.80 |  |
|  | 8319 |  |  | 0.58 |  |
|  | 8330 |  |  | **0.48** |  |
|  | 8331 |  |  | **0.53** |  |
|  | 8332 |  |  | **0.54** |  |
|  | 8339 |  |  | **0.63** |  |
|  | 8340 |  |  | **0.62** |  |
|  | 8343 |  |  | 0.68 |  |
|  | 8357 |  |  | 1.30 |  |
|  | 8359 |  |  | **0.57** |  |
|  | 8361 |  |  | **0.60** |  |
|  | 8363 |  |  | 1.24 |  |
|  | 8365 |  |  | 1.07 |  |
|  | 8367 |  |  | **0.57** |  |
|  | 8368 |  |  | **0.63** |  |
|  | 8370 |  |  | **0.75** |  |
|  | 8371 |  |  | **0.56** |  |
|  | 8373 |  |  | 0.82 |  |
|  | 8374 |  |  | 0.93 |  |
|  | 8378 |  |  | 0.97 |  |
|  | 8380 |  |  | **0.59** |  |
|  | 8381 |  |  | **0.55** |  |
|  | 8383 |  |  | 0.86 |  |
|  | 8384 |  |  | **0.69** |  |
|  | 8386 |  |  | 0.78 |  |
|  | 8387 |  |  | 0.90 |  |
|  | 8390 |  |  | 0.87 |  |
|  | 8392 |  |  | **0.54** |  |
|  | 8393 |  |  | **0.60** |  |
|  | 8395 |  |  | 0.73 |  |
|  | 8396 |  |  | 0.83 |  |
|  | 8397 |  |  | 0.75 |  |
|  | 8398 |  |  | 0.76 |  |
|  | 8399 |  |  | 0.71 |  |
|  | 8406 |  |  | 0.79 |  |
|  | 8424 |  |  | **0.49** |  |
|  | 8425 |  |  | **0.54** |  |
|  | 8426 |  |  | **0.52** |  |
|  | 8427 |  |  | **0.64** |  |
|  | 8432 |  |  | **0.52** |  |
|  | 8434 |  |  | **0.53** |  |
|  | 8435 |  |  | **0.52** |  |
|  | 8436 |  |  | **0.54** |  |
|  | 8437 |  |  | **0.67** |  |
|  | 8439 |  |  | **0.59** |  |
|  | 8443 |  |  | **0.55** |  |
|  | 8445 |  |  | **0.54** |  |
|  | 8447 |  |  | **0.59** |  |
|  | 8449 |  |  | **0.67** |  |
|  | 8450 |  |  | **0.58** |  |
|  | 8474 |  |  | **0.62** |  |
|  | 8476 |  |  | **0.68** |  |
|  | 8478 |  |  | **0.66** |  |
|  | 8481 |  |  | 1.16 |  |
|  | 8483 |  |  | **0.63** |  |
|  | 8484 |  |  | **0.55** |  |
|  | 8486 |  |  | **0.61** |  |
|  | 8487 |  |  | **0.61** |  |
|  | 8489 |  |  | **0.86** |  |
|  | 8490 |  |  | **0.66** |  |
|  | 8495 |  |  | 0.99 |  |
|  | 8497 |  |  | **0.65** |  |
|  | 8498 |  |  | **0.58** |  |
|  | 8500 |  |  | **0.62** |  |
|  | 8501 |  |  | **0.63** |  |
|  | 8503 |  |  | 1.04 |  |
|  | 8504 |  |  | **0.76** |  |
|  | 8505 |  |  | **0.67** |  |
|  | 8510 |  |  | 0.99 |  |
|  | 8512 |  |  | **0.63** |  |
|  | 8513 |  |  | **0.61** |  |
|  | 8515 |  |  | **0.57** |  |
|  | 8516 |  |  | **0.66** |  |
|  | 8518 |  |  | 0.92 |  |
|  | 8519 |  |  | 0.76 |  |
|  | 8520 |  |  | **0.53** |  |
|  | 8524 |  |  | 0.94 |  |
|  | 8525 |  |  | **0.57** |  |
|  | 8526 |  |  | **0.60** |  |
|  | 8527 |  |  | **0.58** |  |
|  | 8528 |  |  | **0.63** |  |
|  | 8529 |  |  | 0.79 |  |
|  | 8530 |  |  | 0.92 |  |
|  | 8531 |  |  | **0.61** |  |
|  | 8534 |  |  | 0.90 |  |
|  | 8541 |  |  | 0.79 |  |
|  | 8542 |  |  | 0.72 |  |
|  | 8564 |  |  | **0.60** |  |
|  | 8565 |  |  | **0.56** |  |
|  | 8566 |  |  | **0.58** |  |
|  | 8567 |  |  | **0.54** |  |
|  | 8572 |  |  | **0.55** |  |
|  | 8574 |  |  | **0.63** |  |
|  | 8575 |  |  | **0.60** |  |
|  | 8576 |  |  | **0.57** |  |
|  | 8577 |  |  | **0.55** |  |
|  | 8579 |  |  | **0.61** |  |
|  | 8586 |  |  | **0.57** |  |
|  | 8588 |  |  | **0.66** |  |
|  | 8589 |  |  | **0.62** |  |
|  | 8590 |  |  | **0.62** |  |
|  | 8591 |  |  | **0.57** |  |
|  | 8593 |  |  | **0.66** |  |
|  | 8594 |  |  | 0.65 |  |
|  | 8601 |  |  | **0.66** |  |
|  | 8602 |  |  | **0.62** |  |
|  | 8603 |  |  | **0.67** |  |
|  | 8606 |  |  | **0.67** |  |
|  | 8607 |  |  | **0.54** |  |
|  | 8638 |  |  | **0.68** |  |
|  | 8640 |  |  | 0.74 |  |
|  | 8642 |  |  | 0.73 |  |
|  | 8648 |  |  | **0.67** |  |
|  | 8651 |  |  | 0.77 |  |
|  | 8652 |  |  | **0.67** |  |
|  | 8654 |  |  | **0.66** |  |
|  | 8655 |  |  | 0.78 |  |
|  | 8658 |  |  | **0.68** |  |
|  | 8662 |  |  | **0.71** |  |
|  | 8663 |  |  | **0.67** |  |
|  | 8665 |  |  | 0.79 |  |
|  | 8666 |  |  | **0.71** |  |
|  | 8668 |  |  | **0.67** |  |
|  | 8669 |  |  | **0.72** |  |
|  | 8670 |  |  | 0.84 |  |
|  | 8674 |  |  | **0.67** |  |
|  | 8677 |  |  | 0.73 |  |
|  | 8678 |  |  | **0.72** |  |
|  | 8680 |  |  | 0.75 |  |
|  | 8681 |  |  | 0.75 |  |
|  | 8683 |  |  | **0.64** |  |
|  | 8684 |  |  | 0.74 |  |
|  | 8685 |  |  | 0.77 |  |
|  | 8686 |  |  | 0.68 |  |
|  | 8690 |  |  | **0.56** |  |
|  | 8693 |  |  | 0.74 |  |
|  | 8694 |  |  | 0.74 |  |
|  | 8696 |  |  | **0.65** |  |
|  | 8697 |  |  | 0.75 |  |
|  | 8699 |  |  | 0.68 |  |
|  | 8700 |  |  | 0.78 |  |
|  | 8701 |  |  | **0.64** |  |
|  | 8704 |  |  | **0.64** |  |
|  | 8705 |  |  | **0.57** |  |
|  | 8708 |  |  | 0.64 |  |
|  | 8709 |  |  | 0.76 |  |
|  | 8711 |  |  | **0.57** |  |
|  | 8712 |  |  | 0.66 |  |
|  | 8714 |  |  | 0.73 |  |
|  | 8715 |  |  | 0.70 |  |
|  | 8716 |  |  | 0.65 |  |
|  | 8719 |  |  | **0.61** |  |
|  | 8720 |  |  | **0.51** |  |
|  | 8732 |  |  | 0.85 |  |
|  | 8756 |  |  | **0.63** |  |
|  | 8757 |  |  | **0.63** |  |
|  | 8758 |  |  | **0.61** |  |
|  | 8767 |  |  | **0.69** |  |
|  | 8768 |  |  | **0.65** |  |
|  | 8770 |  |  | **0.65** |  |
|  | 8771 |  |  | **0.62** |  |
|  | 8772 |  |  | **0.65** |  |
|  | 8781 |  |  | 0.77 |  |
|  | 8782 |  |  | **0.66** |  |
|  | 8783 |  |  | **0.65** |  |
|  | 8784 |  |  | **0.70** |  |
|  | 8785 |  |  | **0.78** |  |
|  | 8786 |  |  | **0.73** |  |
|  | 8787 |  |  | 0.71 |  |
|  | 8795 |  |  | 0.80 |  |
|  | 8796 |  |  | **0.93** |  |
|  | 8797 |  |  | **0.69** |  |
|  | 8798 |  |  | **0.69** |  |
|  | 8800 |  |  | 0.89 |  |
|  | 8801 |  |  | 0.75 |  |
|  | 8802 |  |  | **0.59** |  |
|  | 8808 |  |  | 0.80 |  |
|  | 8809 |  |  | **0.91** |  |
|  | 8810 |  |  | **0.72** |  |
|  | 8812 |  |  | 0.91 |  |
|  | 8813 |  |  | 0.83 |  |
|  | 8814 |  |  | **0.65** |  |
|  | 8816 |  |  | **0.61** |  |
|  | 8825 |  |  | 0.85 |  |
|  | 8826 |  |  | 0.69 |  |
|  | 8849 |  |  | **0.74** |  |
|  | 8851 |  |  | 0.78 |  |
|  | 8853 |  |  | 0.88 |  |
|  | 8860 |  |  | 0.79 |  |
|  | 8861 |  |  | **0.69** |  |
|  | 8863 |  |  | **0.77** |  |
|  | 8864 |  |  | 0.79 |  |
|  | 8866 |  |  | **0.78** |  |
|  | 8867 |  |  | **0.79** |  |
|  | 8874 |  |  | **0.69** |  |
|  | 8877 |  |  | 0.85 |  |
|  | 8878 |  |  | **0.77** |  |
|  | 8880 |  |  | 0.83 |  |
|  | 8881 |  |  | 0.86 |  |
|  | 8882 |  |  | 0.88 |  |
|  | 8884 |  |  | 0.83 |  |
|  | 8885 |  |  | **0.71** |  |
|  | 8886 |  |  | **0.73** |  |
|  | 8889 |  |  | **0.68** |  |
|  | 8892 |  |  | 0.91 |  |
|  | 8893 |  |  | 0.83 |  |
|  | 8895 |  |  | 0.79 |  |
|  | 8896 |  |  | 0.89 |  |
|  | 8897 |  |  | 0.91 |  |
|  | 8898 |  |  | 0.77 |  |
|  | 8899 |  |  | 0.92 |  |
|  | 8900 |  |  | 0.83 |  |
|  | 8901 |  |  | **0.73** |  |
|  | 8902 |  |  | **0.63** |  |
|  | 8903 |  |  | 0.90 |  |
|  | 8905 |  |  | **0.63** |  |
|  | 8908 |  |  | 0.96 |  |
|  | 8909 |  |  | 0.88 |  |
|  | 8911 |  |  | 0.72 |  |
|  | 8912 |  |  | 0.86 |  |
|  | 8913 |  |  | 0.98 |  |
|  | 8914 |  |  | 0.79 |  |
|  | 8915 |  |  | 0.95 |  |
|  | 8917 |  |  | 0.80 |  |
|  | 8918 |  |  | **0.63** |  |
|  | 8919 |  |  | 0.68 |  |
|  | 8920 |  |  | 0.91 |  |
|  | 8921 |  |  | **0.71** |  |
|  | 8923 |  |  | **0.60** |  |
|  | 8926 |  |  | 0.89 |  |
|  | 8927 |  |  | 0.88 |  |
|  | 8930 |  |  | 0.77 |  |
|  | 8931 |  |  | 0.92 |  |
|  | 8932 |  |  | 0.86 |  |
|  | 8936 |  |  | **0.66** |  |
|  | 8937 |  |  | **0.62** |  |
|  | 8938 |  |  | **0.64** |  |
|  | 8940 |  |  | 0.77 |  |
|  | 8942 |  |  | **0.62** |  |
|  | 8945 |  |  | 0.75 |  |
|  | 8946 |  |  | 0.76 |  |
|  | 8948 |  |  | 0.72 |  |
|  | 8949 |  |  | 0.79 |  |
|  | 8950 |  |  | 0.83 |  |
|  | 8954 |  |  | **0.64** |  |
|  | 8955 |  |  | **0.57** |  |
|  | 8958 |  |  | 0.60 |  |
|  | 8962 |  |  | **0.64** |  |
|  | 8964 |  |  | **0.67** |  |
|  | 8970 |  |  | **0.61** |  |
|  | 8973 |  |  | 0.69 |  |
|  | 8977 |  |  | **0.62** |  |
|  | 8978 |  |  | 0.72 |  |
|  | 8993 |  |  | 0.74 |  |
|  | 9003 |  |  | **0.73** |  |
|  | 9005 |  |  | **0.70** |  |
|  | 9006 |  |  | **0.70** |  |
|  | 9007 |  |  | **0.73** |  |
|  | 9017 |  |  | 0.77 |  |
|  | 9019 |  |  | **0.76** |  |
|  | 9020 |  |  | **0.81** |  |
|  | 9021 |  |  | **0.76** |  |
|  | 9022 |  |  | 0.80 |  |
|  | 9031 |  |  | 0.89 |  |
|  | 9033 |  |  | 0.83 |  |
|  | 9034 |  |  | 1.00 |  |
|  | 9035 |  |  | 0.87 |  |
|  | 9036 |  |  | 0.83 |  |
|  | 9044 |  |  | 0.91 |  |
|  | 9046 |  |  | 0.84 |  |
|  | 9047 |  |  | **1.01** |  |
|  | 9048 |  |  | 0.98 |  |
|  | 9049 |  |  | 0.87 |  |
|  | 9050 |  |  | 0.73 |  |
|  | 9054 |  |  | **0.62** |  |
|  | 9063 |  |  | 1.00 |  |
|  | 9064 |  |  | 0.99 |  |
|  | 9065 |  |  | 0.79 |  |
|  | 9068 |  |  | **0.62** |  |
|  | 9078 |  |  | 1.00 |  |
|  | 9079 |  |  | 1.01 |  |
|  | 9080 |  |  | 1.00 |  |
|  | 9082 |  |  | 0.72 |  |
|  | 9092 |  |  | 0.99 |  |
|  | 9101 |  |  | 0.80 |  |
|  | 9103 |  |  | 0.84 |  |
|  | 9112 |  |  | **0.78** |  |
|  | 9115 |  |  | 0.84 |  |
|  | 9116 |  |  | 0.86 |  |
|  | 9117 |  |  | 0.91 |  |
|  | 9118 |  |  | 0.82 |  |
|  | 9138 |  |  | **0.78** |  |
|  | 9141 |  |  | 0.91 |  |
|  | 9142 |  |  | 0.89 |  |
|  | 9143 |  |  | 0.96 |  |
|  | 9145 |  |  | 1.00 |  |
|  | 9146 |  |  | **0.80** |  |
|  | 9147 |  |  | **0.77** |  |
|  | 9154 |  |  | **0.77** |  |
|  | 9155 |  |  | 0.81 |  |
|  | 9157 |  |  | 0.99 |  |
|  | 9158 |  |  | 0.92 |  |
|  | 9159 |  |  | 0.99 |  |
|  | 9160 |  |  | 0.90 |  |
|  | 9162 |  |  | 0.87 |  |
|  | 9163 |  |  | **0.76** |  |
|  | 9164 |  |  | **0.67** |  |
|  | 9170 |  |  | **0.72** |  |
|  | 9171 |  |  | 0.80 |  |
|  | 9173 |  |  | 1.08 |  |
|  | 9174 |  |  | 1.00 |  |
|  | 9175 |  |  | 1.03 |  |
|  | 9176 |  |  | 0.92 |  |
|  | 9178 |  |  | 0.89 |  |
|  | 9179 |  |  | 0.79 |  |
|  | 9180 |  |  | **0.67** |  |
|  | 9181 |  |  | 0.78 |  |
|  | 9182 |  |  | 1.01 |  |
|  | 9183 |  |  | 0.76 |  |
|  | 9188 |  |  | 0.75 |  |
|  | 9189 |  |  | 0.76 |  |
|  | 9191 |  |  | 1.04 |  |
|  | 9192 |  |  | 1.08 |  |
|  | 9193 |  |  | 1.13 |  |
|  | 9194 |  |  | 0.95 |  |
|  | 9197 |  |  | 0.83 |  |
|  | 9198 |  |  | 0.72 |  |
|  | 9199 |  |  | 0.78 |  |
|  | 9200 |  |  | **0.71** |  |
|  | 9201 |  |  | 1.09 |  |
|  | 9202 |  |  | **0.67** |  |
|  | 9203 |  |  | **0.77** |  |
|  | 9208 |  |  | 0.77 |  |
|  | 9209 |  |  | **0.77** |  |
|  | 9212 |  |  | 1.05 |  |
|  | 9213 |  |  | 1.21 |  |
|  | 9214 |  |  | 1.06 |  |
|  | 9218 |  |  | 0.75 |  |
|  | 9219 |  |  | 0.81 |  |
|  | 9220 |  |  | **0.70** |  |
|  | 9223 |  |  | **0.72** |  |
|  | 9224 |  |  | 0.80 |  |
|  | 9229 |  |  | **0.77** |  |
|  | 9231 |  |  | 0.98 |  |
|  | 9232 |  |  | 1.19 |  |
|  | 9233 |  |  | 1.17 |  |
|  | 9237 |  |  | 0.77 |  |
|  | 9238 |  |  | 0.72 |  |
|  | 9240 |  |  | 0.73 |  |
|  | 9241 |  |  | 0.72 |  |
|  | 9248 |  |  | 1.13 |  |
|  | 9249 |  |  | 1.19 |  |
|  | 9252 |  |  | 0.76 |  |
|  | 9253 |  |  | 0.76 |  |
|  | 9256 |  |  | 0.70 |  |
|  | 9279 |  |  | 0.84 |  |
|  | 9283 |  |  | 0.85 |  |
|  | 9297 |  |  | 0.88 |  |
|  | 9298 |  |  | 0.88 |  |
|  | 9310 |  |  | 0.95 |  |
|  | 9311 |  |  | 0.99 |  |
|  | 9323 |  |  | 1.04 |  |
|  | 9324 |  |  | 1.12 |  |
|  | 9338 |  |  | 1.02 |  |
|  | 9339 |  |  | 1.21 |  |
|  | 9345 |  |  | 0.79 |  |
|  | 9354 |  |  | 0.97 |  |
|  | 9359 |  |  | 1.00 |  |
|  | 9360 |  |  | 0.82 |  |
|  | 9365 |  |  | 0.88 |  |
|  | 9368 |  |  | 0.94 |  |
|  | 9372 |  |  | 0.99 |  |
|  | 9373 |  |  | 1.01 |  |
|  | 9377 |  |  | 0.72 |  |
|  | 9383 |  |  | 1.00 |  |
|  | 9398 |  |  | 0.91 |  |
|  | 9402 |  |  | 0.94 |  |
|  | 9413 |  |  | 1.02 |  |
|  | 9437 |  |  | 0.94 |  |
|  | 9439 |  |  | 0.92 |  |
|  | 9440 |  |  | 0.93 |  |
|  | 9442 |  |  | 1.02 |  |
|  | 9443 |  |  | 0.91 |  |
|  | 9449 |  |  | **0.74** |  |
|  | 9451 |  |  | 0.88 |  |
|  | 9452 |  |  | 0.91 |  |
|  | 9453 |  |  | 1.07 |  |
|  | 9455 |  |  | 1.12 |  |
|  | 9456 |  |  | 0.93 |  |
|  | 9457 |  |  | 0.82 |  |
|  | 9463 |  |  | **0.73** |  |
|  | 9466 |  |  | 0.89 |  |
|  | 9467 |  |  | 0.90 |  |
|  | 9468 |  |  | 1.08 |  |
|  | 9471 |  |  | 1.03 |  |
|  | 9472 |  |  | 0.84 |  |
|  | 9473 |  |  | 0.83 |  |
|  | 9474 |  |  | 1.01 |  |
|  | 9476 |  |  | 0.77 |  |
|  | 9480 |  |  | 0.80 |  |
|  | 9483 |  |  | 1.02 |  |
|  | 9484 |  |  | 0.87 |  |
|  | 9485 |  |  | 1.03 |  |
|  | 9488 |  |  | 1.17 |  |
|  | 9489 |  |  | 0.93 |  |
|  | 9490 |  |  | 0.77 |  |
|  | 9491 |  |  | 1.06 |  |
|  | 9494 |  |  | 0.78 |  |
|  | 9495 |  |  | 0.82 |  |
|  | 9499 |  |  | 0.88 |  |
|  | 9502 |  |  | 1.04 |  |
|  | 9503 |  |  | 0.87 |  |
|  | 9504 |  |  | 0.95 |  |
|  | 9505 |  |  | 0.92 |  |
|  | 9508 |  |  | 0.99 |  |
|  | 9509 |  |  | 0.82 |  |
|  | 9510 |  |  | 0.92 |  |
|  | 9511 |  |  | 0.86 |  |
|  | 9513 |  |  | 0.82 |  |
|  | 9514 |  |  | 0.87 |  |
|  | 9515 |  |  | 0.82 |  |
|  | 9519 |  |  | 0.90 |  |
|  | 9520 |  |  | 0.86 |  |
|  | 9521 |  |  | 0.91 |  |
|  | 9522 |  |  | 0.88 |  |
|  | 9523 |  |  | 0.94 |  |
|  | 9524 |  |  | 0.86 |  |
|  | 9528 |  |  | 0.81 |  |
|  | 9529 |  |  | 0.90 |  |
|  | 9530 |  |  | **0.82** |  |
|  | 9532 |  |  | 0.81 |  |
|  | 9533 |  |  | 0.90 |  |
|  | 9534 |  |  | 0.85 |  |
|  | 9538 |  |  | 0.83 |  |
|  | 9539 |  |  | 0.90 |  |
|  | 9541 |  |  | 0.86 |  |
|  | 9542 |  |  | 0.89 |  |
|  | 9543 |  |  | 0.81 |  |
|  | 9547 |  |  | 0.90 |  |
|  | 9548 |  |  | 0.86 |  |
|  | 9550 |  |  | 0.85 |  |
|  | 9551 |  |  | 0.83 |  |
|  | 9559 |  |  | 0.79 |  |
|  | 9562 |  |  | 0.83 |  |
|  | 9563 |  |  | 0.87 |  |
|  | 9566 |  |  | 0.77 |  |
|  | 9594 |  |  | 0.91 |  |
|  | 9604 |  |  | 0.93 |  |
|  | 9673 |  |  | 0.85 |  |
|  | 9682 |  |  | 1.00 |  |
|  | 9700 |  |  | 0.94 |  |
|  | 9704 |  |  | 0.88 |  |
|  | 9705 |  |  | **0.84** |  |
|  | 9707 |  |  | 0.89 |  |
|  | 9708 |  |  | 0.89 |  |
|  | 9739 |  |  | 1.03 |  |
|  | 9750 |  |  | 1.09 |  |
|  | 9775 |  |  | 0.87 |  |
|  | 9789 |  |  | **0.77** |  |
|  | 9791 |  |  | 0.96 |  |
|  | 9792 |  |  | 0.88 |  |
|  | 9799 |  |  | 0.92 |  |
|  | 9802 |  |  | 0.88 |  |
|  | 9803 |  |  | 0.95 |  |
|  | 9807 |  |  | **0.74** |  |
|  | 9809 |  |  | 0.96 |  |
|  | 9810 |  |  | 0.93 |  |
|  | 9811 |  |  | 0.93 |  |
|  | 9817 |  |  | 0.87 |  |
|  | 9820 |  |  | 0.92 |  |
|  | 9821 |  |  | 0.95 |  |
|  | 9822 |  |  | 0.93 |  |
|  | 9828 |  |  | 0.94 |  |
|  | 9829 |  |  | 0.94 |  |
|  | 9834 |  |  | **0.75** |  |
|  | 9835 |  |  | 0.88 |  |
|  | 9839 |  |  | 0.96 |  |
|  | 9840 |  |  | 0.94 |  |
|  | 9846 |  |  | 0.93 |  |
|  | 9847 |  |  | 0.94 |  |
|  | 9852 |  |  | 0.84 |  |
|  | 9855 |  |  | 0.93 |  |
|  | 9856 |  |  | 0.92 |  |
|  | 9952 |  |  | 0.93 |  |
|  | 9953 |  |  | 0.93 |  |
|  | 9966 |  |  | 0.88 |  |
|  | 9967 |  |  | 0.88 |  |
|  | 9979 |  |  | 0.69 |  |
|  | 10406 | MAG | Ester | 0.99 |  |
|  | 10407 |  |  | 0.98 |  |
|  | 10408 |  |  | 0.99 |  |
|  | 10409 |  |  | **0.92** |  |
|  | 10410 |  |  | 0.99 |  |
|  | 10411 |  |  | 1.00 |  |
|  | 10412 |  |  | 1.01 |  |
|  | 10413 |  |  | 1.02 |  |
|  | 10414 |  |  | 0.72 |  |
|  | 10415 |  |  | 0.91 |  |
|  | 10416 |  |  | 0.91 |  |
|  | 10417 |  |  | 1.03 |  |
|  | 10418 |  |  | 0.94 |  |
|  | 10419 |  |  | 1.03 |  |
|  | 10420 |  |  | 0.96 |  |
|  | 10421 |  |  | 0.77 |  |
|  | 10422 |  |  | 0.98 |  |
|  | 10423 |  |  | 0.99 |  |
|  | 10424 |  |  | 0.91 |  |
|  | 10425 |  |  | 1.00 |  |
|  | 10426 |  |  | 0.97 |  |
|  | 10427 |  |  | 0.98 |  |
|  | 10428 |  |  | 0.88 |  |
|  | 10429 |  |  | 1.04 |  |
|  | 10430 |  |  | 0.90 |  |
|  | 10431 |  |  | 0.93 |  |
